# Harnessing Endogenous Stimuli for Responsive Materials in Theranostics

**DOI:** 10.1021/acsnano.0c09115

**Published:** 2021-02-08

**Authors:** Alexander B. Cook, Paolo Decuzzi

**Affiliations:** Laboratory of Nanotechnology for Precision Medicine, Istituto Italiano di Tecnologia, Via Morego 30, 16163 Genova, Italy

**Keywords:** materials, nanoparticles, responsive polymers, nanomedicine, biological stimuli, formulations, pH, enzymes

## Abstract

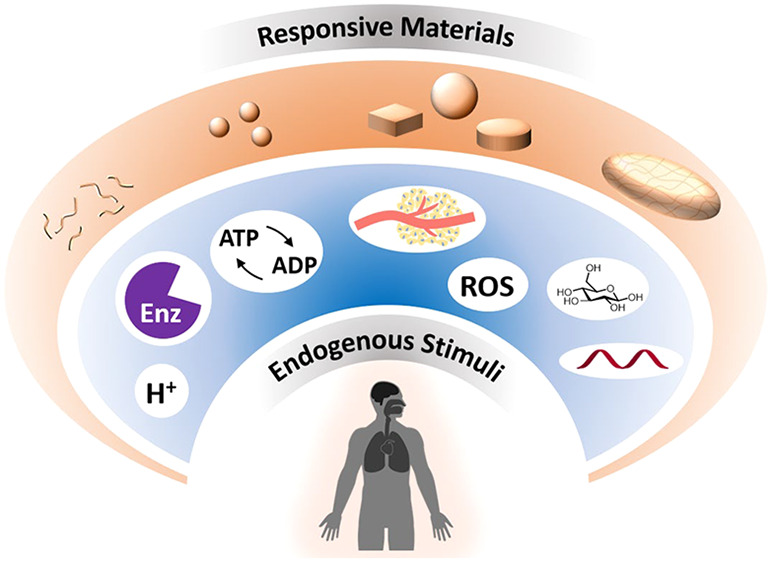

Materials that respond to endogenous stimuli are being leveraged to enhance spatiotemporal control in a range of biomedical applications from drug delivery to diagnostic tools. The design of materials that undergo morphological or chemical changes in response to specific biological cues or pathologies will be an important area of research for improving efficacies of existing therapies and imaging agents, while also being promising for developing personalized theranostic systems. Internal stimuli-responsive systems can be engineered across length scales from nanometers to macroscopic and can respond to endogenous signals such as enzymes, pH, glucose, ATP, hypoxia, redox signals, and nucleic acids by incorporating synthetic bio-inspired moieties or natural building blocks. This Review will summarize response mechanisms and fabrication strategies used in internal stimuli-responsive materials with a focus on drug delivery and imaging for a broad range of pathologies, including cancer, diabetes, vascular disorders, inflammation, and microbial infections. We will also discuss observed challenges, future research directions, and clinical translation aspects of these responsive materials.

Advances in materials science for treating, imaging, and sensing diseases have received tremendous attention over the past few decades.^1,2^ In particular, nanoscale materials and particles that can help drugs have higher efficacies and better target diseased sites have become prominent areas of research.^3^ Originally, this concept was proposed in the 1960s, when lipids and polymers started to be used to formulate drugs and develop products with an understanding of how to modulate pharmacokinetics, pharmacodynamics, biocompatibility, and the material–biology interface.^4^ The approach has allowed devices and therapies to be developed which can help to both regulate the dosing and monitor the disease.^5^ The ultimate aim is to get drugs and imaging agents where and when they are needed inside the body. Utilizing materials with defined responses to biological signals from the body and pathological abnormalities is an elegant and effective strategy to achieve this.^6−10^

Stimuli-responsive materials for these applications vary in both composition and physical size and shape. Specific material characteristics and related administration routes depend on the disease, but all require consideration of complex biological variables to achieve precision release or activation.^11,12^ On the macroscopic side, device coatings are being developed to provide stimuli-responsive antibacterial activity, and precisely engineered microscale materials are increasingly useful as highly tunable, implantable depot-type devices. At the other end of the size range, nanoparticles have for some time been the focus for a large number of researchers looking to merge therapy and diagnosis—theranostics. These nanoparticle materials can be metallic, inorganic, lipid-based, or polymeric in nature. Lipid-based nanoparticles have had the most success in reaching the clinic, with the liposomal doxorubicin formulations Doxil and Myocet and the recently approved solid lipid nanoparticle siRNA formulation Onpattro being good examples.^13,14^ On the molecular scale, individual polymer conjugates incorporating therapeutics, imaging agents, or both are possibly the most versatile systems but remain synthetically challenging. The research teams of Ringsdorf, Kopeček, and Duncan pioneered the field of pharmaceutical polymer conjugates.^15−17^ Controlling the physical morphological properties of biomedical materials improves the chances of the active agent reaching the diseased site.^18^ To improve release of the active agent over the correct time scale, certain stimuli responses are able to be harnessed. The triggers can be either internal or external signals. External stimuli can be thermal, ultraviolet (UV) light, near-infrared (NIR) radiation, magnetic fields, or ultrasound.^19^ Synthetic materials responsive to internal stimuli offer the chance to attempt to mimic the elegant mechanisms of natural biological systems. These innate biological stimuli can include pH, redox, reactive oxygen species (ROS), glucose, adenosine triphosphate (ATP), hypoxia, and many types of enzymes.^20^

Beyond the increasing complexity in nanomedicine synthesis, attention is also being paid to incorporating imaging modalities into the one-dosage form.^21^ This merging of therapeutics and diagnostics makes it possible to combine imaging and treatment strategies that allow biodistribution of the nanocarriers to be assessed, the treatment mechanism of action to be determined, and disease progression to be monitored in real-time. Imaging techniques accessible for theranostic applications include positron emission tomography (PET), magnetic resonance imaging (MRI), optical/fluorescent imaging, ultrasonography, computed X-ray tomography (CT), and single-photon emission computed tomography (SPECT).^22,23^ Typically these techniques require imaging contrast agents (paramagnetic metal ions, fluorescent/near-infrared probes, radionuclides) and thus careful consideration of the best materials or nanoparticles to achieve maximum contrast. The application of endogenous stimuli-responsive imaging agents *in vivo* allows enhancement of the signal-to-noise ratio in the targeted tissue environment compared to the surrounding normal tissue. The improvement in temporal control of theranostic imaging resolution allows the detection of smaller lesions and abnormalities which are undetectable with traditional methods.^23,24^ Hence, this combination of theranostics with bioresponsive materials can improve longitudinal investigations that monitor disease changes in response to treatment, providing information about disease progression.^25−27^

In this Review, we will first outline the design strategies for endogenous stimuli-responsive materials and how these can help achieve spatiotemporal control in both therapy and diagnostic imaging. We then highlight a selection of literature examples of endogenous stimuli-responsive materials using various biological markers such as enzymes, pH, redox, hypoxia, glucose, and ATP. The focus will be on particularly important historical examples of stimuli-responsive materials and selected important recent contributions, to give a broad but comprehensive overview. A range of applications will be discussed, including cancer, cardiovascular disease, arthritis, brain disorders, diabetes, and bacterial infections. Finally, we will mention some aspects relating to clinical translation of stimuli-responsive theranostic systems and future directions. A number of reviews have previously covered bioresponsive materials^3,6^ and stimuli-responsive materials in theranostics,^24,28^ which are recommended; however, here the focus is on harnessing endogenous stimuli to enhance temporal control over drug release or diagnostic imaging.

## Spatial Control of Theranostic Systems

**Figure 1:**
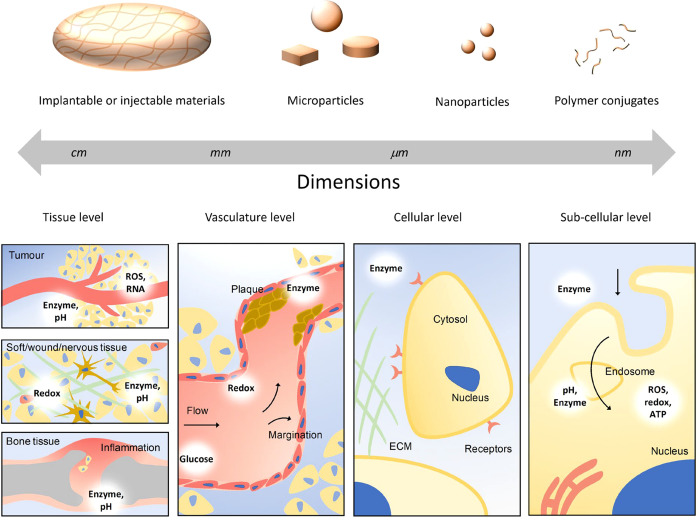
Schematic showing design synergies between materials’ physiochemical properties and biological environments. Biomaterials varying in shape and size from centimeters to nanometers can be applied in tissue/cell environments having structural features across similar length scales, while also expressing various cues able to be recognized by endogenous stimuli-responsive materials (including enzymes, pH, redox, glucose, hypoxia, ATP, and nucleic acids).

Tuning the rate or location of drug delivery in the body helps to reduce adverse effects to large doses of therapeutics.^29−31^ High doses are required in non-formulated or non-targeted systems in order to overcome pharmacological inefficiencies, but those come with increased toxicity or other side effects. Delivery systems of all sizes for drugs and imaging agents help avoid these problems by increasing spatial control (Figure 1). For macroscale systems this can be achieved through using local administration routes combined with materials or devices to retain the active agents at a particular site.^1,2^ Another route to achieve spatial control is through particle design parameters such as size, shape, and stiffness, which can help particulate systems avoid immune recognition and localize to certain organs.^11,32^ The ideal design approach would begin with a product profile with the desired target pathology and efficacy defined; this would then determine how to proceed when choosing a route of administration and thus formulation dimension characteristics (conjugate, nanoparticle, microparticle, or macroscale depot). Use of targeting ligands combined with particulate systems has also proven to be effective for active targeting of particular tissues or cells.^33^

### Local Delivery Systems

Macroscale and local delivery systems avoid problems related to systemic delivery such as serum proteases, serum protein adsorption, renal clearance, and infusion/hypersensitivity reactions.^34,35^ Macroscale devices or formulations can range from injectable hydrogels and coatings on implantable devices to transdermal patches.^36,37^ Depot systems can include active ingredient reservoirs surrounded by a physical barrier or agent dispersed through a stable matrix material. Early examples included semi-permeable membranes, like the porous silicon membrane used to slowly deliver anesthetic gases, developed by Folkman,^38^ while more modern matrix-type devices avoid possible problems from reservoir failure and rapid toxic drug release.^39^ Hydrogel matrices can be fabricated from natural or synthetic polymers, with active agent release usually controlled through diffusion. Diffusion can be tuned either by altering mesh pore size, drug-complex size and/or non-bonding interactions with the gel, polymer molecular weight between cross-links, and number of cross-links or by introducing stimuli response. Hydrogels have been shown to be effective for a number of different applications. In ocular drug delivery, thermoresponsive polymers have been used to form biocompatible gel depots in the eye of animals with a model of glaucoma^40^ and other ocular inflammation-related diseases.^41−43^ An injectable hydrogel was used by Silva and Mooney to improve the angiogenic treatment of ischemic diseases with spatiotemporal controlled delivery of the vascular endothelial growth factor (VEGF).^44^ The gel formulation induced increased angiogenesis in the hindlimbs of mice in a preclinical model of ischemia, compared to bolus delivery. Intramuscular injections of drug suspension are also used for extended release of therapeutics. Olanzapine can be administered this way, with the product Zyprexa Relprevv. Existing medical devices can be integrated with therapeutics through methods such as device coatings to provide additional functionality, such as the Surmodics Bravo system.^45^ Therapeutics and diagnostic agents can also be incorporated into devices such as stents through direct encapsulation in the device material.^46^

### Particle-Based Delivery Systems

Encapsulating or conjugating theranostic agents to particle delivery systems which can then be used for intravenous injections offers benefits in a variety of clinical settings where larger macroscale or local delivery systems are not necessary.^12^ Designing particles to improve localization in a particular tissue can be achieved through careful consideration of parameters such as size, shape, surface chemistry, and mechanical stiffness.^11,12,47−49^

The vast majority of nanomedicines and nanotheranostics developed in the recent past are based on spherical nanoparticles (inorganic and metal nanoparticles, polymeric particles, lipid nanoparticles, liposomes). Improving the biodistribution of these systems is often achieved with active targeting ligands, including peptides such as the rabies viral glycoprotein (RVG),^50^ nuclear localization signal peptide (NLS),^51^ and GRKKRRQRRRPQ in the TAT peptide present in human immunodeficiency virus (HIV).^52^ Antibody- and sugar-derived targeting molecules featured in are also prominent strategies.^53^ Modulation of particle stiffness has shown to offer elements of spatial control of particles for drug delivery and theranostics. The authors have shown that the deformability of microparticles of controlled size and shape can help determine how particles are recognized by the immune system and also their distribution in certain organs.^54,55^ Caruso and colleagues showed that the extent of particle deformability can highly influence their interactions with biological environments.^56,57^ Engineering non-spherical or anisotropic delivery systems has been shown to influence localization and vascular margination.^58−60^ Mitragotri and co-workers developed polystyrene nanoparticles of various shapes and showed that rod-shaped particles had higher cell uptake and transcytosis of intestine cells compared to spherical nanoparticles.^61−63^ Discoidal polymeric nanoconstructs can efficiently marginate in vasculature, which can offer improvements for thrombolytic therapies.^58^ Long anisotropic particles such as tubular polymersomes,^64,65^ peptide nanofibers,^66−69^ and carbon nanotubes^70^ can also make significant differences in cell uptake biodistribution and treatment efficacy.

## Temporal Control of Theranostics through Endogenous Stimuli

Stimuli response of materials and programmed release of agents can be achieved through the incorporation of functional moieties into formulations or devices. These functional motifs are designed to have biological sensitivity.^6^ Such variations in physiological parameters can be markers of certain diseased states, *e.g.*, cancer, degenerative diseases, cardiovascular diseases, or inflammation, and provide a means to achieve temporal control of drug release or diagnostic probe switch-on.^21^ These triggers can be found at different scales and in different regions in the body, from the tissue level through to vasculature and cellular/intracellular levels (Figure 1). Depending on the progression of the disease, these triggers can impart temporal control on the therapeutic and imaging intervention. Indeed, the expression of inflammatory cytokines and enzymes in inflamed joints, the concentration of metalloproteases in primary and metastatic malignant masses, or the levels of glucose during hypo-/hyperglycemia can all vary over time as the disease progresses. The ideal endogenous stimuli-responsive system would take into account size, shape, surface chemistry, and stiffness characteristics of the material and optimize these for the particular target and administration route.^71−75^

**Figure 2:**
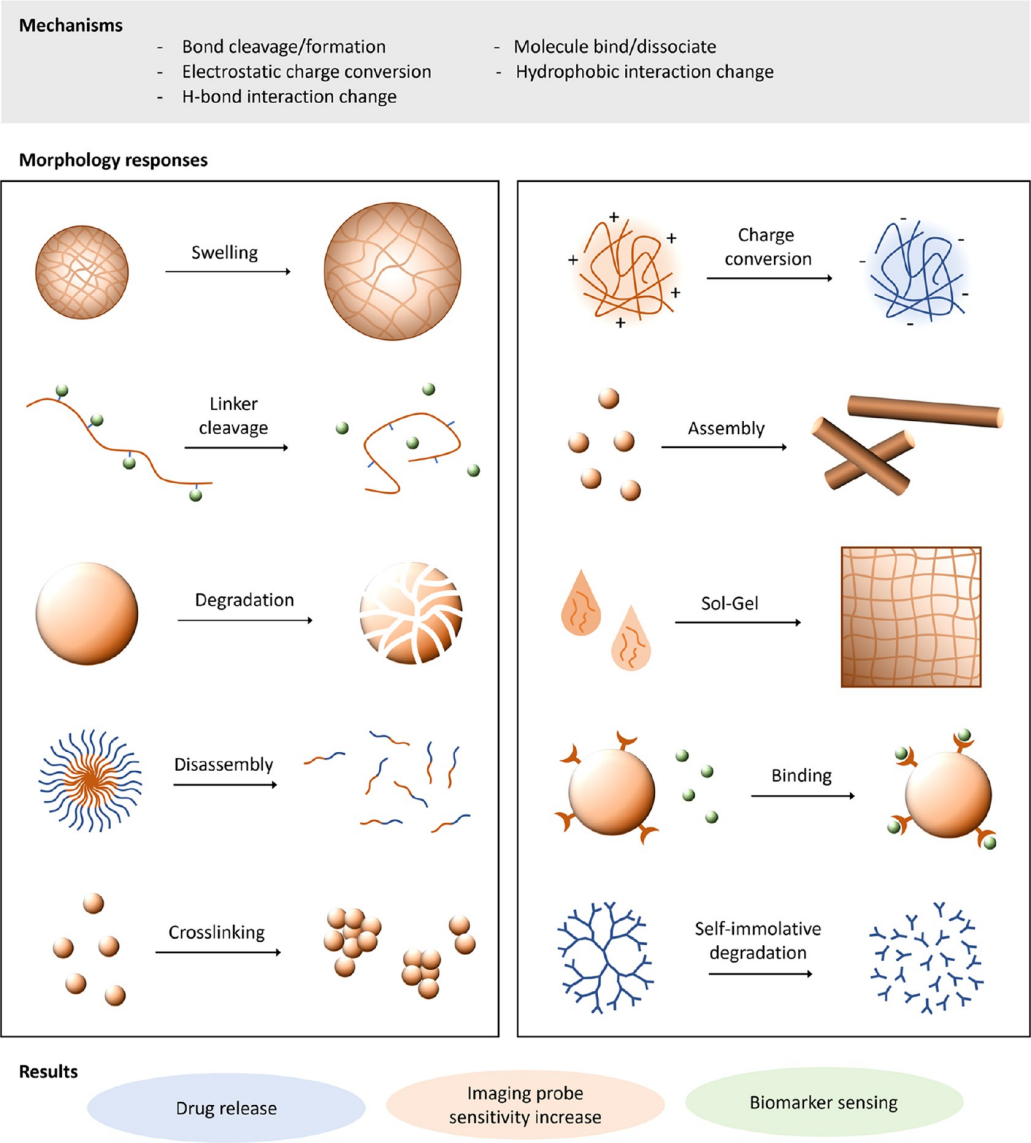
Material response to endogenous stimuli trigger can be diverse. Response can be due to changes in covalent bonding, electrostatic charge, H-bonding interactions, or other intermolecular interactions, leading to morphology changes in materials from polymer conjugates to nanoparticles, self-assembled systems, or hydrogels. These materials’ response mechanisms have seen applications in drug release, biomarker sensing, and activatable imaging agents.

On exposure to biological stimuli, there are a number of differing mechanisms that can achieve the associated change in material morphology or properties. These are summarized in Figure 2, and include bond cleavage/formation, charge conversion, hydrophobic interaction, H-bonding, and guest/host molecule binding/dissociation. The induced morphology change then triggers the desired action in response to the particular biological signal/environment. In theranostics, this can be material morphology or charge switch for increased permeation or cell-uptake, drug release, imaging agent release and switch-on, biomarker binding, and diagnostic switch-on. This section highlights the most common variations of endogenous stimuli-responsive materials for theranostic applications, organized by particular stimulus.

Temperature differences have also been used extensively to give responsive materials for various applications, the majority of which have utilized exogenous application of temperature enhancement through combination with radiation/photothermal techniques.^76,77^ Endogenous temperature differences in pathologies due to enhanced metabolism rates or inflamed tissues have also been reported but have been reviewed elsewhere.^78,79^

## Enzyme Response

Expression of enzymes such as proteases and lipases can be upregulated or downregulated in certain pathologies like cancer and inflammation, and therefore can be used to achieve enzyme-sensitive agent release or triggering in the desired target.^26,27^ The enzymes used for this bioresponsive behavior can be extracellular or localized within certain subcellular organelle.^80^ A major advantage of these materials is the highly specific nature of enzyme-mediated material response, which allows the targeting of precise tissues or diseases. A summary of enzyme-responsive materials for these applications is shown in Table 1. Design strategies for these enzyme-responsive materials focus on a few key mechanisms, mostly involving peptide bond cleavage: (i) cleavable drug linkers (covalent), (ii) cleavable bonds in nano-/microparticle carriers allowing (non-covalently bound) drug release, (iii) cleavable linkers altering surface charge or functionality, and (iv) cleavable bonds inducing particle assembly/morphology changes.

**Table 1:** Examples of Endogenous Stimuli-Responsive Materials Triggered by Enzymatic Activity, Used in Drug Delivery and Imaging Applications

enzyme	endogenous stimuli-responsive chemical group	therapy/diagnostic application
MMPs	GCRD-GPQGIWGQ-DRCG, GCRD-GPQGIAGQ-DRCG	BMP-2-containing hydrogels for bone tissue regenerative medicine^82^
	KCGGYRGCK	enzyme-responsive hydrogel for cell biology, drug delivery, and regenerative medicine^83^
	GPLGIAGQ	enzyme-responsive tumor-targeting therapy^84^
	GPLGVRGK	materials with improved tumor accumulation and penetration^85^
	GPLGLAGGWGERDGS	enzyme-responsive particle size increase for retention of drug delivery vehicles in tumors^86^
	PLGLAG	tumor microenvironment removal of particle hydrophilic shielding sequence EK6 and exposure of the cell-penetrating sequence R8^87^
	GPLGVRGC	activatable fluorescence for imaging-guided nanoparticle-based photothermal therapy^88^

plasmin	GGKFKTGG	responsive release of morphogens in vascularized osteogenesis^89^
	GCYKNRDCG	hydrogel system for enzyme-responsive *in vivo* regeneration of critical-sized bone defects^90^
	AFK	enzyme-responsive integrin-targeted plasmin-cleavable doxorubicin prodrug^91^

thrombin	FPipRS	endogenously stimuli-responsive hydrogel material for autoregulated anti-coagulation^92^
	G(DF)PRGFPAGG	anti-thrombotic hydrogel system with thrombin-responsive fibrinolytic activity^93^

cathepsins	GFLG	combination therapy for hormone-dependent cancer^95^
		enzyme-cleavable chemotherapeutic drug–polymer conjugates^100^
		polymer conjugates for the treatment of ovarian cancer with non-invasive fate monitoring^101^
		FRET-based imaging in ovarian tumors^102^
		AIE and phototoxicity of enzyme-activatable theranostic^103^
	FKFL	degradable cationic polymers for DNA release^96^
	GGGF, PMGLP	enzyme-selective improvement of diagnostic and radiotherapeutic drug delivery systems for pancreatic cancer^97^
	GFLGKGLFG	degradable prodrug-based nanomedicine containing nifuroxazide and doxorubicin against breast cancer^98^
	VC	auristatin monoclonal antibody conjugates for cancer therapy^104^
		antibiotic–antibody conjugate against *Staphylococcus aureus*(105)
		enzyme-cleavable polymeric ciprofloxacin prodrug for alveolar pulmonary infections^106^

trypsin	GRRRGK	hydrogels for protein delivery targeted to the small intestine^107^
		nanogels with siRNA for lowering macrophage TNF-α in inflammatory bowel disease^108^

esterase	esters	nucleic acid-functionalized nanocapsule-based enzyme-responsive small-molecule release^109^
		mesoporous silica nanoparticles with stimuli-responsive doxorubicin release with esterase^110^
		theranostic FRET probe nanoparticles for intracellular imaging in cancer^111^

lipase	esters	wound-healing scaffold with lipase-activatable ciprofloxacin-based prodrug and a diagnostic probe^112^
		lipase-triggered drug release system for multimodal anti-fungal therapy^113^

elastase	CGAAPVRGGGC	human neutrophil elastase-sensitive hydrogel for controlled release of proteins^114^
	AA	mussel-inspired adhesive hydrogels^115^

phosphatase	RGD*p*S, *p*YRGD*p*S	phosphatase-responsive surfaces with property changes in the presence of enzymes/cells^116^
	Fmoc-pY	polymer bioconjugates with phosphatase enzyme and thermal responsiveness^117^

lysyl oxidase	cysteamine	lysyl oxidase-responsive strain-stiffening PEG hydrogels mimicking extracellular matrix mechanisms^118^

γ-glutamyl transpepidase	γ-glutamylamide	charge-switching camptothecin–polymer conjugate for enhanced tumor cell transcytosis and penetration^119^

caspase	CDVEDIETDPra	fluorescent probe responsive to two caspase activities in living cells (monitoring of apoptotic process)^120^
	DEVDPra	theranostic platinum prodrug AIE apoptosis sensor^121^

**Figure 3:**
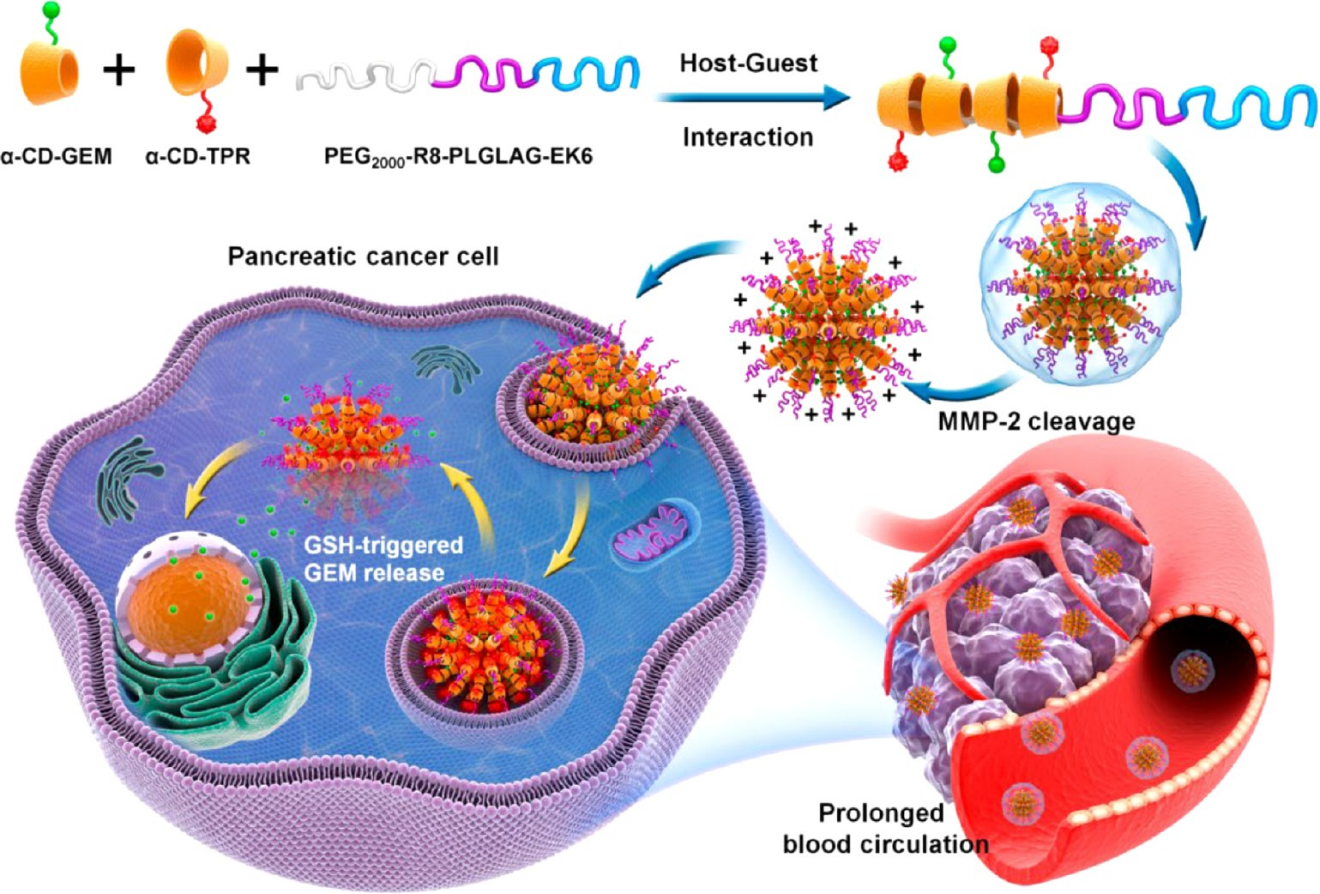
Study of supramolecular aggregation-induced emission nanodots, with MMP responsiveness for image-guided cancer treatment. Nanoparticles formed *via* host–guest assembly and multiple stages of tumor-microenvironment enzymatic activation and GSH-triggered drug release are shown schematically. Reproduced with permission from ref (87). Copyright 2020 American Chemical Society.

Matrix metalloproteinases (MMPs) are a widely used group of enzymes to induce material response in theranostic applications.^81^ The use of MMP degradable peptide sequences in synthetic materials was shown by the group of Hubbell in the early 2000s for tissue engineering applications.^82^ They showed that the poly(ethyleneglycol) (PEG)–peptide hydrogels formed through Michael addition of PEG vinyl sulfones and the peptide’s terminal cysteine groups were able to be degraded by cell-secreted MMPs, allowing cell invasion. Further hydrogel materials have been established by Anseth and co-workers, where MMP-responsive extracellular matrix mimics were created using a facile thiol–ene photopolymerization mechanism.^83^ Incorporating MMP degradable peptide sequences into nanoparticle drug carriers and nanoparticle-based contrast agents has been shown by a number of research groups.^84−86^ In the field of theranostics, MMP-responsive systems are very promising. Aggregation-induced emission (AIE) nanodots have been used for image-guided drug delivery.^87^ These supramolecular nanoparticle systems were formed from the assembly of functional cyclodextrins conjugated to the anti-cancer therapeutic gemcitabine with MMP-2-sensitive PEG–peptide polymers. The particles have a zwitterionic stealth peptide shell (alternating glutamic acid and lysine residues (EK6)), which helps achieve longer circulation times, and when in the tumor microenvironment with overexpressed MMP-2, the shell is cleaved, exposing a cell-penetrating peptide for enhanced tumor cell uptake. *In vivo* fluorescence imaging showed that this improved tumor tissue accumulation, and orthotopic and subcutaneous pancreatic cancer mouse models showed improved effects of the anti-cancer therapeutic by much reduced BxPC-3 tumor growth over 18 days (Figure 3). Zhao *et al*. showed that gold nanorods could be used to create a MMP-responsive theranostic platform able to be used for fluorescence imaging-guided photothermal therapy.^88^ An asymmetric cyanine fluorescent probe was attached to the gold particle surface through a GPLGVRGC peptide linker, which allowed selective cleavage and thus imaging agent switch-on only in tumor tissue. Precision photothermal therapy was then performed at the tumor site specifically with 808 nm wavelength irradiation. SCC-7 tumor-bearing mice were treated with image-guided photothermal therapy at 4 h post injections, leading to subsequent tumor growth after 14 days being lowest for the MMP-responsive system.

**Figure 4:**
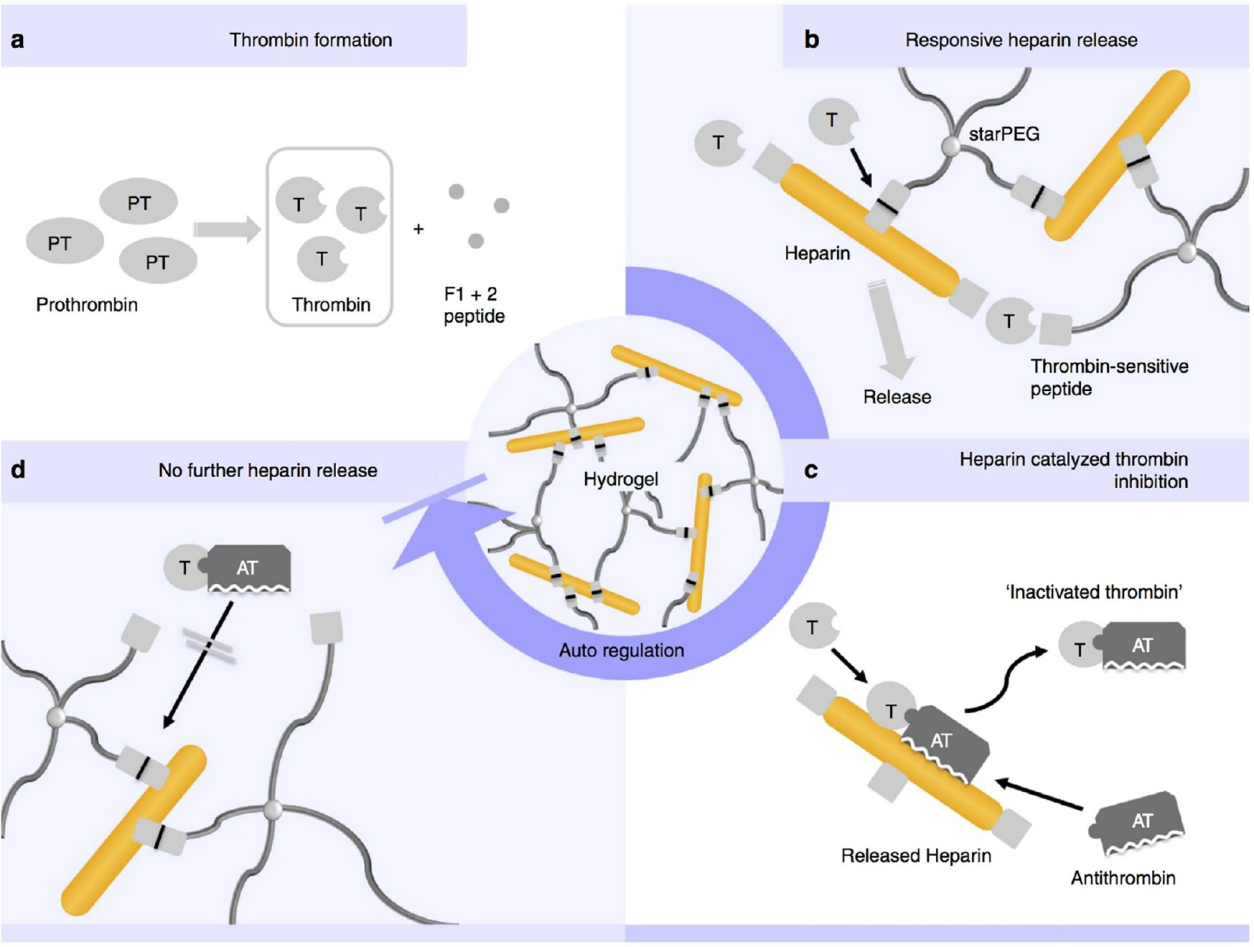
Thrombin-responsive PEG-based heparin hydrogels used in the treatment of coagulation-related diseases. (a) Thrombin generation from prothrombin. (b) Selective peptide linker cleavage, releasing heparin. (c) Heparin-catalyzed thrombin deactivation. (d) Gel degradation halted on inactivation of thrombin in a self-regulated release mechanism. Reproduced with permission from ref (92). Copyright 2013 Springer Nature.

The serum proteases thrombin and plasmin have been employed as stimuli for responsive drug delivery materials for a number of different applications. The Segura group demonstrated plasmin-responsive nanocapsules containing two proteins, VEGF and PDGF, that were able to be tuned to release their cargo at different rates, depending on the amount and chirality of the cleavable peptide linker incorporated into the formulation.^29^ Peptide–polymer hybrid nanoparticles were fabricated by Kader *et al*. for the plasmin-responsive release of bone morphogenetic protein-2 (BMP2) and VEGF.^89^ The target was for the particles to be degraded in the presence of human mesenchymal stem cells and human endothelial colony-forming cells to trigger osteogenesis and vasculogenesis for bone regeneration, which was assessed *in vitro*. Plasmin-sensitive hydrogels^90^ and drug conjugates^91^ have also been shown to be promising enzyme-responsive materials. On the other hand, thrombin-responsive materials are effective in temporal-controlled anti-thrombotic treatments, including for ischemic strokes and other coagulation-related conditions.^92,93^ Maitz *et al*. demonstrated a responsive PEG-based hydrogel system with a built-in closed-loop response mechanism (Figure 4).^92^ The material responded to blood coagulation-generated thrombin, which released the anti-coagulant heparin as a therapeutic agent to treat thrombosis or emboli.

**Figure 5:**
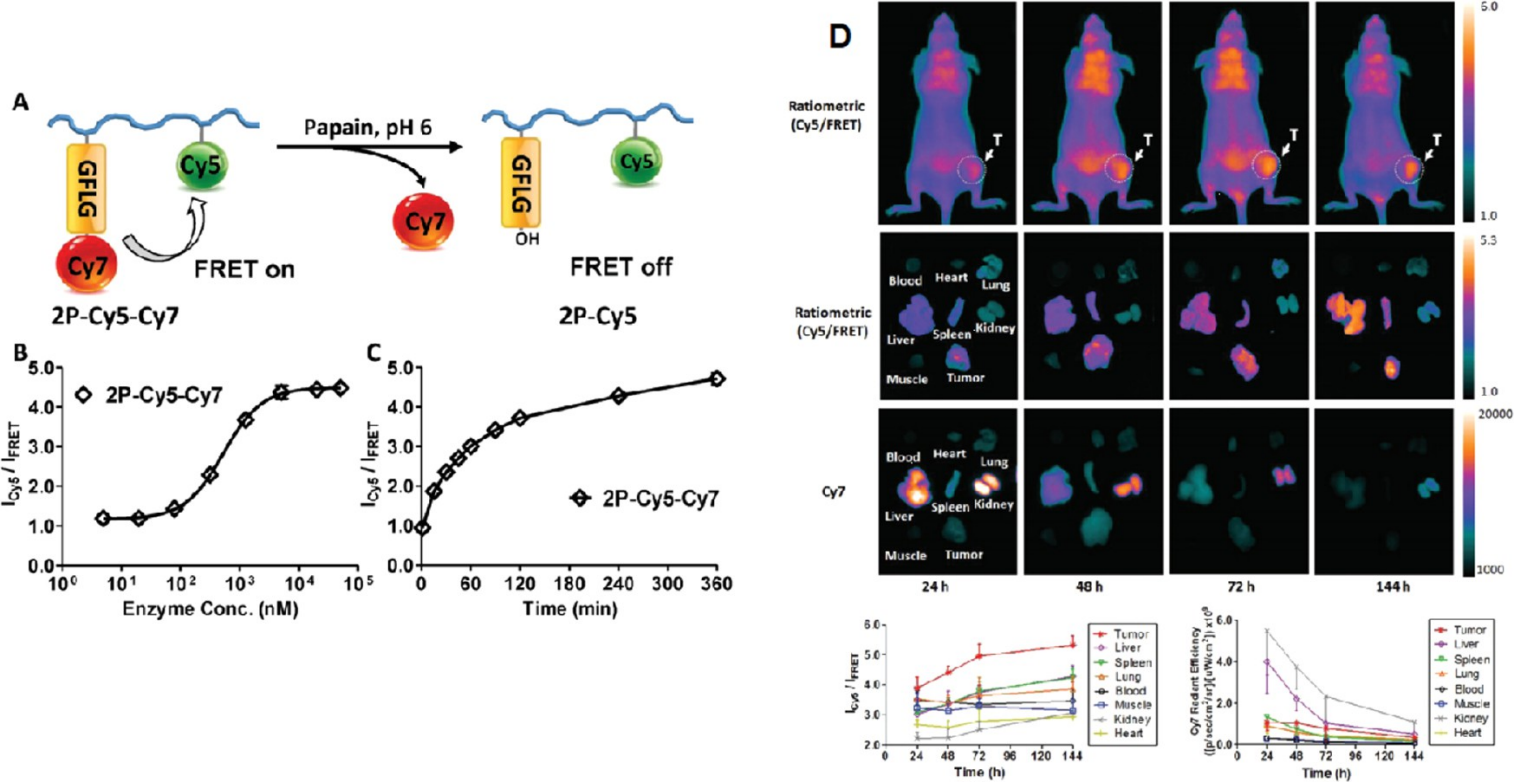
Application of cathepsin B in theranostic responsive systems. (A) Designed FRET imaging response of polymer conjugate on exposure to enzyme cathepsin B (papain). (B) Cy5 FRET ratio of conjugate when incubated with different concentrations of papain. (C) Cy5 FRET ratio change over time (4 h with 5 × 10^–6^ M papain). (D) *In vivo* FRET imaging of mice bearing A2780 human ovarian tumor after intravenous administration of polymer conjugate. Reproduced with permission from ref (102). Copyright 2017 Wiley VCH.

The cathepsin class of enzymes comprises important proteases for responsive polymer–drug conjugates and prodrugs with endosomal activation. Cysteine cathepsins (B, C, F, H, K, L1, L2/V, O, S, W, X/Z) are located in the endosome/lysosome intracellular compartment, making them useful in the design of enzyme-degradable nanomaterials, conjugates, biomaterials, and probes for intracellular release or activation.^94−98^ In particular, cathepsin B has been well studied for cancer applications, including breast, lung, prostate, and colorectal cancers, due to its overexpression in tumor tissue cells.^94,99^ Kopeček and Duncan, among others, have had success with cathepsin-cleavable linkers between drug molecules and polymers, research which started in the 1980s and progressed to phase II clinical trials for anti-cancer therapy.^16,17,100^ This work is based on the amino acid sequence GFLG, which is cleaved by cathepsin B, between doxorubicin and poly(*N*-(2-hydroxypropyl)methacrylamide (pHPMA). More recently, the Kopeček group combined the polymer prodrugs with advanced imaging modalities including radioisotope ^125^I^101^ and Förster resonance energy transfer (FRET) dye pair Cy5 and Cy7^102^ to obtain labeled enzyme-responsive conjugates for theranostics. The conjugate pHPMA-GFLG-epirubicin had much improved pharmacokinetics with higher molecular weight polymers (33.2 h half-life), and tumor remission and long-term tumor growth inhibition were achieved in a mouse xenograft model of human ovarian carcinoma.^101^Figure 5 shows the pHPMA conjugates with cathepsin B-cleavable linkers for *in vitro* and *in vivo* FRET imaging.^102^ The results indicated that a higher concentration of cathepsin B in tumor cells is able to trigger release of FRET imaging agents inside cells, which is also confirmed with mice bearing human ovarian tumors. The much lower concentration of cleaved polymer prodrug in healthy tissues suggests this could be an excellent strategy for increasing circulation half-life and minimizing off-target effects of toxic small-molecule drugs. The GFLG cathepsin B linker was also employed by Liu and Tang for enzyme-activatable AIE and image-guided photodynamic therapy (PDT).^103^ Their combined diagnostic and therapeutic probe contained an orange AIE fluorogen, peptide-linked hydrophilic moieties, and cyclic arginine–glycine–aspartate (cRGD) integrin-targeting units. The hydrophilic nature of the probe changed to hydrophobic upon linker cleavage by cathepsin B, causing the AIE probe aggregation and allowing PDT to generate ROS for tumor ablation.

Another type of cathepsin-cleavable linker is the simple valine–citrulline (VC) linker. This enzyme-responsive strategy has been used by Seattle Genetics in their commercial ADCETRIS antibody–drug conjugate for intracellular-specific drug release.^104^ The same VC linker has been used by Genentech for enzyme-activatable antibody–antibiotic conjugate treatment of infections.^105^ A Seattle-based collaboration led by Stayton also used the VC linker but in polymer ciprofloxacin prodrugs for the targeted treatment of intracellular bacterial infections in lung alveolar macrophages.^106^ Incorporation of a mannose-based monomer into the polymer chains increased water solubility and targeting to alveolar macrophages. In a mouse model of pneumonic tularemia, the polymeric prodrug was able to achieve 100% survival, compared to 0% survival using the free drug.

Enzymatic degradation can also be used to target larger organs such as the intestines. Knipe and Peppas showed that poly(methacrylic acid-*co*-*N*-vinylpyrolidone) microgel particles cross-linked with a trypsin-degradable GRRRGK peptide cross-linker were able to be loaded with the protein insulin for oral delivery routes.^107^ The peptide cross-linked hydrogels were stable in rat gastric fluid yet degraded in rat intestinal fluid, demonstrating the organ-targeted carrier degradation. The authors also investigated these materials to encapsulate siRNA-containing nanogels for specific TNF-α knockdown in the intestine.^108^ Microgels were proposed to be protective of their nanogel cargo in the harsh conditions of the stomach and allowed enzymatically triggered release in the intestine. Nanogels loaded with siRNA were able to be uptaken by RAW 264.7 macrophages and showed 40% TNF-α knockdown compared to the non-treatment control, illustrating their potential for treatment of inflammatory bowel disease.

Esterases are also important enzymes used to improve temporal control in both drug delivery and theranotstic systems.^109−111^ Stoddart and co-workers used mesoporous silica nanoparticles loaded with doxorubicin, which were capped with poly(β-amino ester), to allow responsive drug release in the presences of porcine liver esterase through degradation of the polymer capping agent backbone.^110^ The particles were investigated for antiproliferative activity against MDA-MB-231 human breast cancer cells. Saxena *et al*. harnessed the specificity of esterases for development of a responsive theranostic FRET probe.^111^ An amphiphilic l-amino acid polymer formed self-assembled nanoparticles smaller than 200 nm in size, encapsulating both the green fluorescent drug curcumin and the red fluorescent probe Nile red to give FRET activity. The particles were stable in the extracellular environment (red fluorescence) and were degraded by lysozymal esterases intracellularly, which then turned off the FRET as the probes became too distant. The released curcumin was demonstrated to be toxic to breast cancer cells *in vitro*.

**Figure 6:**
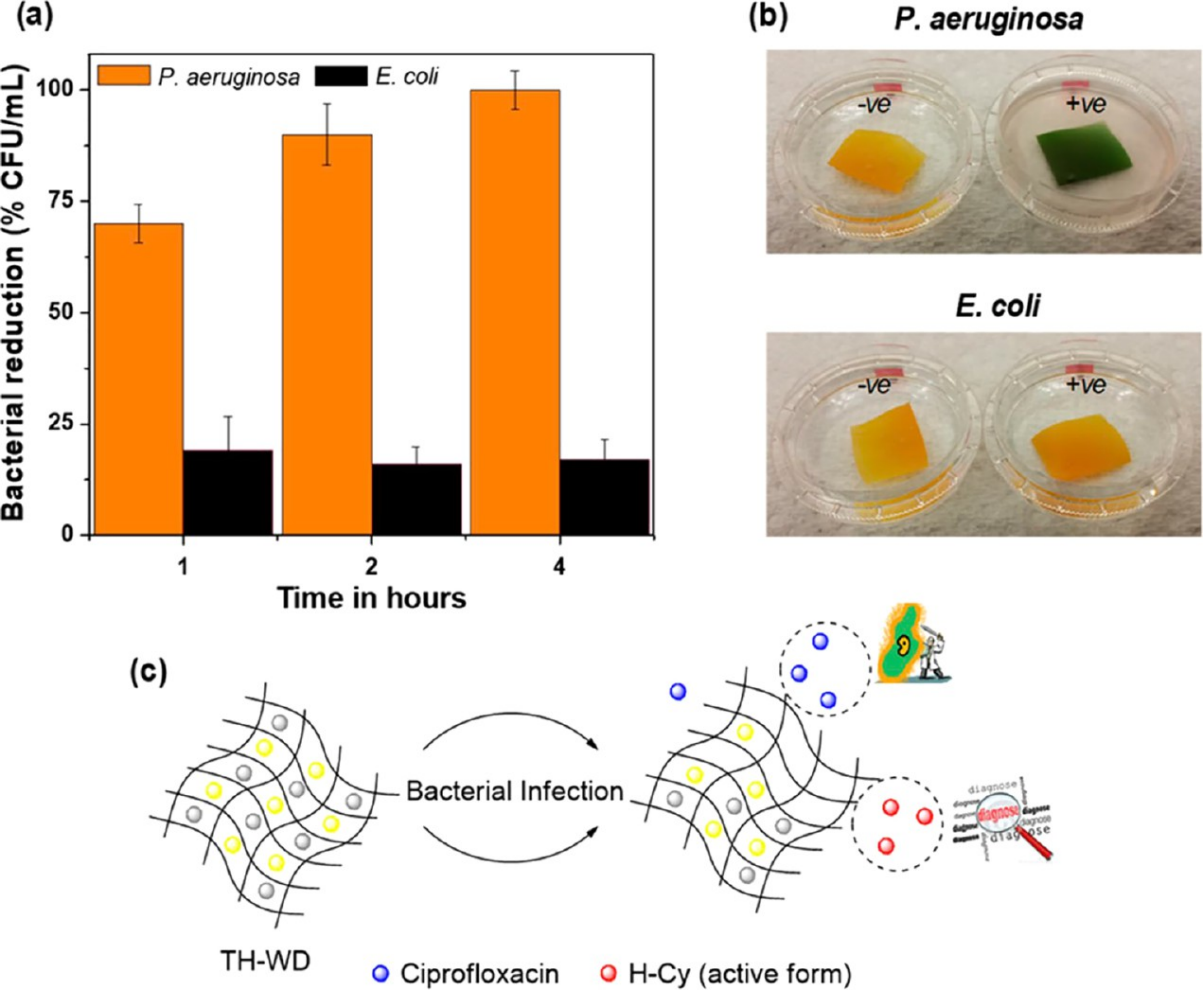
Application of lipase in theranostic responsive systems. (a) Antibacterial properties of a lipase-responsive theranostic wound dressing system against *P. aeruginosa* and *E. coli* over time. (b) Color change after 4 h incubation with bacteria. (c) Schematic of the enzyme activation system. Reproduced with permission from ref (112). Copyright 2019 American Chemical Society.

**Figure 7:**
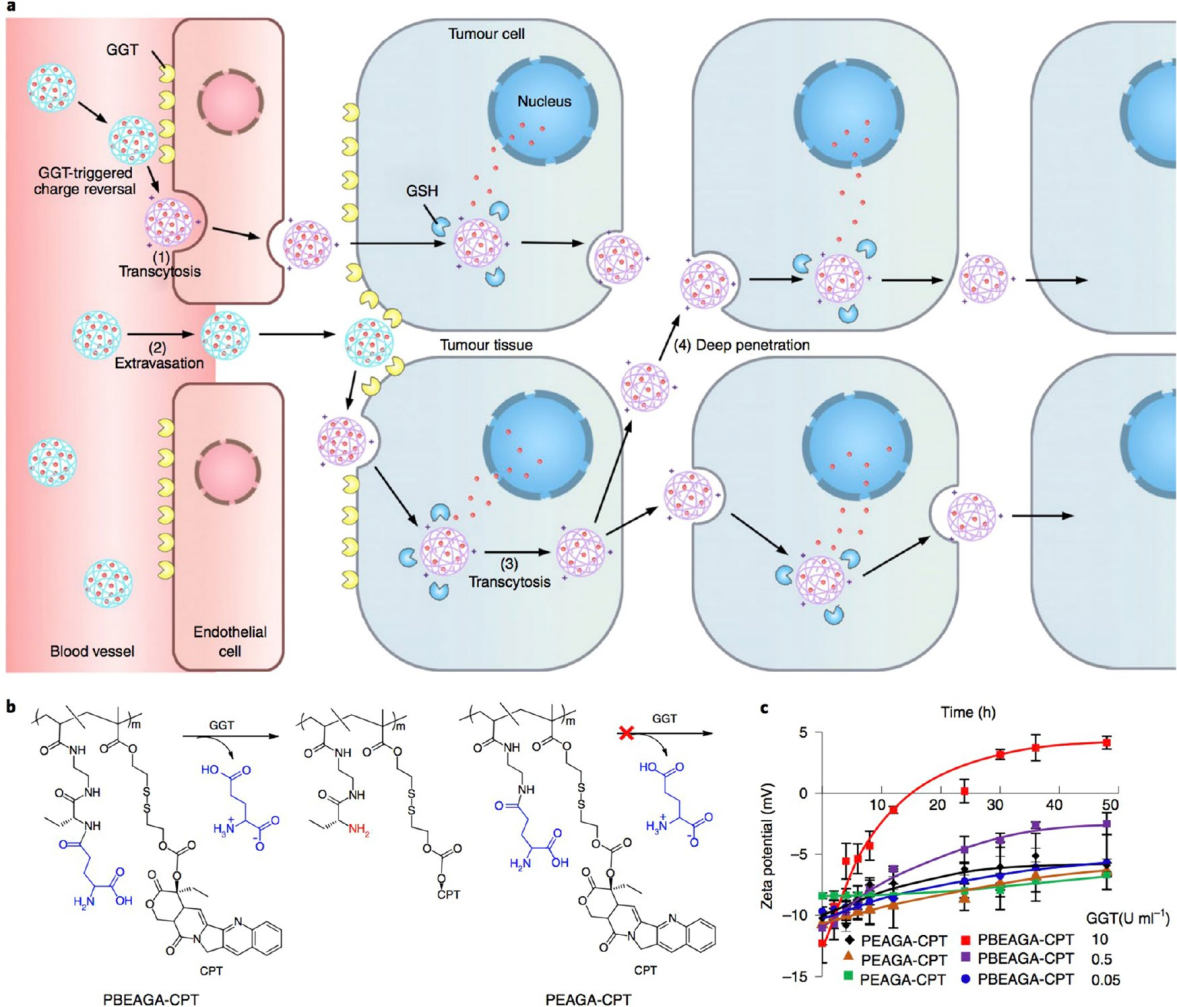
Design strategy for an enzyme-responsive polymer–drug conjugate which forms 5–10 nm assemblies. (a) Schematic illustrating the proposed polycation-induced transcytosis tumor penetration. (b) Conjugate chemical structure and charge-switching responsive behavior. (c) Zeta potential changes on exposure to membrane γ-glutamyl transpeptidase. Reproduced with permission from ref (119). Copyright 2019 Springer Nature.

To a lesser extent, a number of additional enzymes have been utilized to trigger material responses in theranostic and drug delivery systems, such as lipases,^112,113^ elastases,^114,115^ phosphotases,^116,117^ lysyl oxidase,^118^ γ-glutamyl transpeptidase,^119^ and caspases.^120,121^ Theranostic systems for monitoring/treating wounds and wound infections is a growing area of research in which materials science can play an important role. Lipases have been used to provide a responsive material for *Pseudomonas aeruginosa*-specific diagnostic and infection inhibition (Figure 6).^112^ Electrospun fibers of polyurethane formed a scaffold in which a prodrug of ciprofloxacin and a chromogenic probe were loaded. Both the prodrug and the probe were only activated after exposure to the high level of lipases expressed by *P. aeruginosa* (100% ± 4% reduction of *P. aeruginosa* (ATCC 27853) within 4 h of contact); with low lipase-producing bacteria or fibroblasts, no toxicity was observed. An *ex vivo* pig skin model of infected burns showed a visible color change over 4 h and a reduction in bacterial load of 87% ± 3% after 4 h of incubation. An important study utilizing γ-glutamyl transpeptidase was reported by the groups of Shen and Gu (Figure 7).^119^ This enzyme is overexpressed on exterior surfaces of endothelial cells and also metabolically active tumor cells bordering blood vessels. The researchers designed a camptothecin–polymer conjugate which undergoes a charge reversal from negatively charged (approximately −10 mV) to positively charged (approximately +5 mV), which enables improved caveolea-mediated endocytosis and transcytosis to achieve deep tumor penetration. In a pancreatic tumor mouse model, the enzyme-responsive conjugate was able to eradicate small solid tumors and extend median survival times from 32 days (control) and 50 days (gemcitabine) to over 75 days post inoculation, where 80% of treated mice still survived.

## Local pH Variations

**Figure 8:**
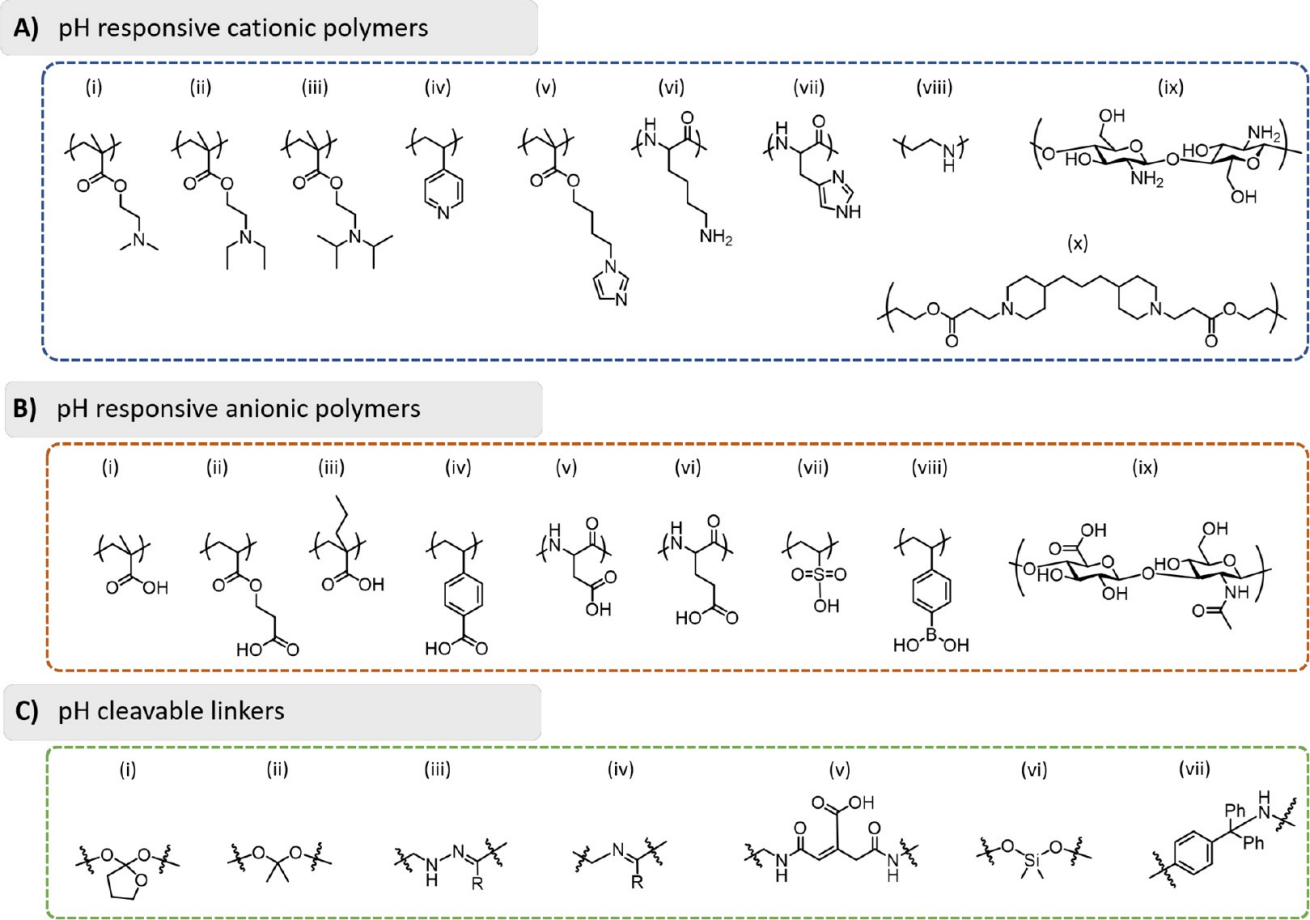
Summary of the functional groups and linkers used in the development of pH-sensitive materials. (A) pH-responsive cationic polymers: (i) poly(2-dimethylaminoethyl methacrylate) (PDMAEMA), (ii) poly(2-diethylaminoethyl methacrylate) (PDEAEMA), (iii) poly(2-diisopropylaminoethyl methacrylate) (PDPAEMA), (iv) poly(4-vinylpyridine) (PVP), (v) poly(4-(1*H*-imidazol-1-yl)butyl methacrylate (PImBuMA), (vi) poly(lysine) (PLys), (vii) poly(histidine) (PHis), (viii) poly(ethylenimine) (PEI), (ix) chitosan, and (x) poly(β-amino ester) (PbAE). (B) pH-responsive anionic polymers: (i) poly(acrylic acid) (PAA), (ii) poly(2-carboxyethyl acrylate) (PCEA), (iii) poly(2-propylacrylic acid) (PPAA), (iv) poly(4-vinylbenzoic acid) (PVBA), (v) poly(aspartic acid) (PAsA), (vi) poly(glutamic acid) (PGA), (vii) poly(vinylsulfonic acid) (PVSA), (viii) poly(vinylphenylboronic acid) (PVPBA), and (ix) hyaluronic acid. (C) pH-cleavable linkers: (i) *ortho*-esters, (ii) ketals/acetals, (iii) hydrazones, (iv) imines, (v) maleic acid amide derivatives (including *cis*-aconityl shown), (vi) silyl ethers, and (vii) trityl derivatives.

Materials that are engineered with groups that respond to changes in pH can undergo changes such as swelling, degradation, dissociation, or lipid membrane disruption. The chemical moieties that enable this usually involve amines or carboxylic acids and cause either bond cleavages or ionizable charge changes (Figure 8). Local pH variations can be indicative of various pathologies and also intracellular locations. For example, the acidification gradient during the endocytosis process from approximately neutral extracellularly to a pH of 6.0–6.8 in early endosomes and finally reaching pH values as low as 5 in lysosomes can be harnessed to allow intracellular imaging agent or drug release. In addition, the tumor microenvironment and also localized inflammatory sites can have significantly lower pH than the surrounding healthy tissue, making tissue targeting of theranostics possible. The classes of materials incorporating these types of pH response can range from polymer conjugates to inorganic and polymer nanoparticles, hydrogels, liposomes and lipid nanoparticles, and supramolecular systems. This section will summarize noteworthy examples of theranostics responsive to pH, focusing on the mentioned classes of materials.

**Figure 9:**
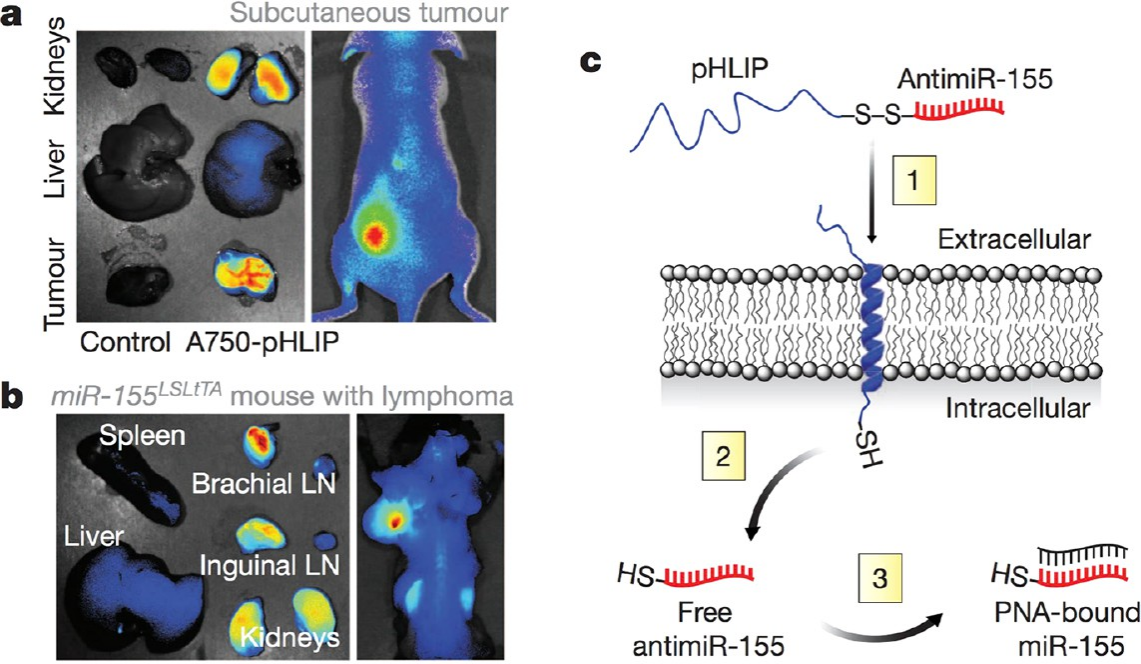
Tumor microenvironment-targeted delivery of antimiRs using a peptide with a low-pH-induced transmembrane structure (pHLIP). Distribution of pHLIP labeled with Alexa Fluor 750, 36 h after systemic administration to (a) nude mouse with miR-155 flank tumors and (b) mouse with lymphadenopathy. (c) Schematic of pHLIP-mediated antimiR delivery. Reproduced with permission from ref (128). Copyright 2015 Springer Nature.

Hydrophilic polymer conjugates offer a versatile and non-complicated strategy to increase the molecular weight, hydrodynamic radius, stability, and circulation half-life of both imaging agents and therapeutics. Acid-sensitive linkers can be easily incorporated by using particular monomers or through chain-end modifications.^122,123^ Fréchet and colleagues introduced pH-responsive acetal linkers in 2004,^124^ initially as cleavable bonds for PEG conjugates of small-molecule drugs and later as linkers for siRNA conjugates with dextran.^125^ By varying the chemical structure of the conjugation from acyclic to cyclic acetals, the release of siRNA from dextran could be tuned from a half-life of 2 h at pH 5 and 120 h at pH 7.4 to multiple days at both pH 5 and 7.4. Polymer conjugates have also proven to be successful for nucleic acid delivery by a number of other research teams. Kataoka and co-workers developed a pH-responsive siRNA conjugate which was anionic at neutral pH and cationic at endosomal pH, causing membrane disruption and increased escape to the cytosol.^126^ Rozema *et al*. reported a delivery system named dynamic polyconjugates, which includes a cationic polymer PBAVE shielded by pH-sensitive PEG units that detach under acidic endosomal conditions, allowing endosome membrane destabilization.^127^ The dynamic polyconjugate technology developed by MirusBio was acquired by Roche and then Arrowhead Pharmaceuticals, which took the system through to phase II clinical trials. Another pH-sensitive RNA conjugate was developed by Slack and colleagues, which is shown in Figure 9.^128^ This platform helps deliver a particular type of microRNA called antimiR through use of a peptide with a low-pH-induced transmembrane structure (pHLIP) which can cross membranes in the acidic tumor environment and release the nucleic acid intracellularly with glutathione redox stimuli. Labeling the conjugate with Alexafluor 750 allows theranostic tracking of the distribution and localization of the material *in vivo*; it localizes to subcutaneous flank model tumors of lymphoid origin and also in a model of disseminated lymphadenopathy. In the subcutaneous model, the conjugate reduced tumor volumes to ∼500 mm^3^ after 6 days, compared to 1500 mm^3^ in the negative control, while survival times increased from 6 to 11 days. The authors note that a limitation of this conjugate delivery system is the need for neutral-charged cargo.

The polymer architecture of conjugate-based theranostic delivery systems can also be varied and allows for introduction of more functional groups for modification and different nanoscale morphologies. Polymer conjugates can range from linear^129,130^ to bottle brush,^131^ branched,^132,133^ and dendritic–linear hybrid polymer architectures.^134−136^ Linear polymers were used in combination with hydrazine linkages for paclitaxel and docetaxel controlled release^130^ and to improve the signal-to-noise ratio in PET fluoromisonidazole imaging in acidic tumor microenvironments.^129^ Highly branched and multifunctional polymer conjugates (with sizes of approximately 10 nm) have been pioneered by Thurecht *et al*. for theranostic applications.^132,133^ The researchers used a synthesis protocol combining PEGMA, the main component, with Cy5-MA, an EGDMA cross-linker, and a hydrazide monomer for pH-responsive drug conjugation, with a peptide aptamer attached to the branched polymer exterior. This system allowed incorporation of many components for tumor-targeted drug delivery and fluorescent imaging *in vivo*, with the optimized targeted polymer HBP-5 having higher accumulation in the tumor tissue and also significant retention at the tumor site due to the targeting ligand–receptor interaction.

Employing polymers as building blocks for endogenous stimuli-responsive theranostics can potentially lead to additional hurdles for regulatory approval. In particular, regulatory authorities can find it difficult to assess materials having a molecular weight dispersity (*i.e.*, not a single molecular entity), although this dispersity could actually offer benefits for avoiding recognition by certain biological processes and immune systems. Use of lipid-based delivery systems (liposomes and lipid nanoparticles) can get around this possible limitation by using single molecular species. Liposomes are possibly the most well-established of nanomedicines in the clinic, but they are less established in stimuli-responsive theranostic intervention.^137,138^ Various researchers have investigated pH-responsive liposomes as carriers of paramagnetic metal ions for MRI. Løkling *et al*. showed that pH-sensitive liposomes could give an increase in longitudinal relaxivity of Gd contrast agents in acidic environments using a dipalmitoylphosphatidylethanolamine (DPPE)/dipalmitoylglycerosuccinate (DSPG) liposome formulation, and that formulation with a small proportion of DSPE-PEG could increase blood circulation half-life.^139−141^ Terreno and co-workers employed a similar strategy but with the pH-sensitive and fusogenic lipid 1-palmitoyl-2-oleoyl-*sn*-glycero-3-phosphoethanolamine (POPE), which gave a formulation for imaging drug delivery by acid-sensitive theranostic release.^142^ Such pH-sensitive liposomes have also been utilized for nucleic acid delivery^143^ and theranostic anti-cancer treatments in murine hepatic carcinoma cell xenograft models with paclitaxel and NIR agent.^144^ More recently, lipid nanoparticles have been making a big impact in both clinical translation and academic research directions.^145,146^ The use of ionizable (*i.e.*, pH-responsive) lipids is the key to this technology and allows high loading of nucleic acid therapeutics at low pH through ionic complexation, neutral charge at pH 7.4 during *in vivo* circulation, and interaction with endosomal membranes at lower pH to facilitate endosome escape. A large selection of publications have highlighted how the chemical structures of the ionizable lipids^147−150^ and their proper integration into the lipid nanoparticles can greatly affect nucleic acid transfection efficacies.^151,152^ It is thought that the p*K*_a_ of the ionizable amines (and therefore their chemical structure) in such lipid systems affects intracellular trafficking pathways. Gilleron *et al*. incorporated imaging agents to investigate this, including gold nanoparticles for enhanced transmission electron microscopy (TEM) imaging contrast, and the fluorescent dye Alexa647 for optical microscopy.^153^ They elegantly showed that escape of siRNA from the endosome to cytosol occurs with an efficiency of between 1 and 2% and happens during early and late endosome periods of cellular uptake. In another study, Patel *et al*. employed a series of CRISPR-induced genetic perturbations of the endosome/lysosome pathway to investigate mechanisms of intracellular delivery of mRNA with ionizable lipid nanoparticles.^154^ By modulating the mTOR late endosome/lysosome signaling pathway, intracellular delivery could be deduced to rely significantly on late endosome/lysosome formation. Indeed, by co-formulating bioactive lipid leukotriene antagonists, which enrich endosomal/lysosomal compartments, intracellular delivery could be increased 2-fold *in vivo* and 3-fold *in vitro*. Other notable recent examples of ionizable lipid nanoparticle formulations include selective organ targeting through lipid formulation,^155^ mRNA delivery for human CAR T cell engineering,^156^ and investigations of human skin explants as a lipid nanoparticle formulation screening strategy.^157^

Supramolecular materials offer the chance to incorporate pH responsivity into particles formed with intermolecular forces and therefore able to disassemble for clearance by the body.^158−160^ Theranostic systems based on supramolecular chemistry have been extensively studied by the group of Xiaoyuan Chen.^161,162^

**Figure 10:**
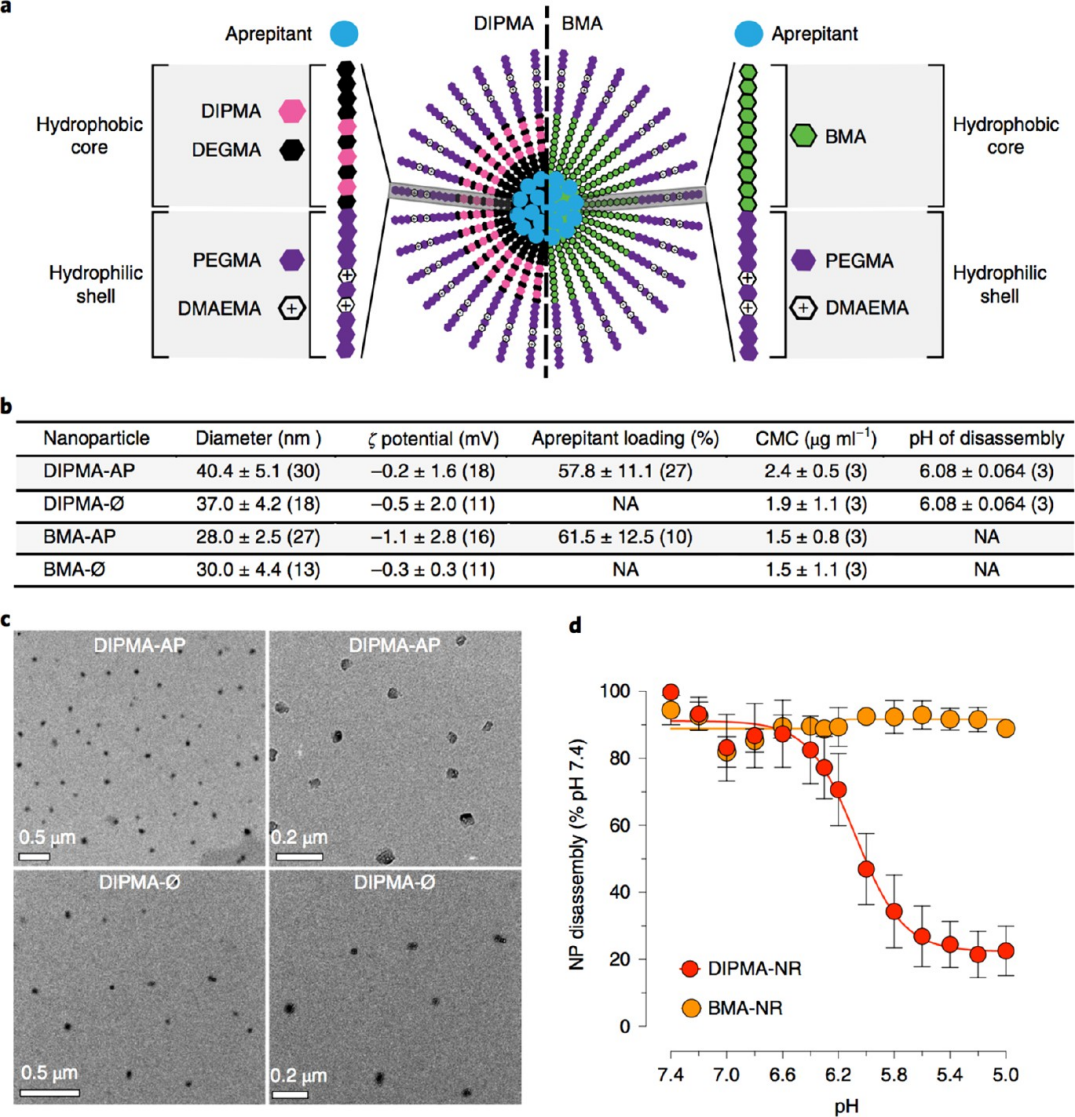
Application of pH-responsive polymer nanoparticles in pain management. (a) Schematic of pH-responsive polymer nanoparticles (from P(PEGMA-*co*-DMAEMA) shell blocks and P(DIPMA-*co*-DEGMA) or P(BMA) core-forming blocks) which target the neurokinin 1 receptor in endosomes to treat chronic pain. (b) Particle characterization data. (c) Particle TEM images. (d) Particle pH-responsive behavior characterization. Reproduced with permission from ref (175). Copyright 2019 Springer Nature.

Polymer nanoparticles are a widely employed class of materials for pH-responsive materials due to their simple production and ease of incorporation of different functional groups and conjugation chemistries.^163−166^ pH-responsive polymer particles were reported by Gaus and Gooding to deliver doxorubicin to the nucleus of MCF7 cells. The authors also conjugated a nuclear localization signal peptide to the surface of the particles to increase nucleus accumulation, while using pair correlation microscopy to reveal mechanistic details about intracellular transport of nanoparticles.^51^ Stayton and colleagues developed an endosomolytic block copolymer nanoparticle system for the delivery of siRNA to the cellular cytosol.^167,168^ By incorporating specific amounts of cationic monomer (DMAEMA), hydrophobic monomer (BMA), and a pH-responsive monomer propyl acrylic acid (PAA), the polymers could complex nucleic acids to form nanoparticles and have pH-dependent membrane disruption activity for enhanced endosomal escape. This system was used to delivery immunostimulatory oligonucleotides with an unmethylated cytosine–phosphate–guanine sequence that mimics the structure of sequences found in bacterial and viral DNA, for use as nanoparticle vaccines.^167^ In addition, a wide variety of cationic polymer nanoparticles complexing nucleic acids have likewise been studied for their pH responsiveness and gene transfection efficiencies.^169−172^ The group of Gao has elegantly shown that pH-responsive block copolymer particles can be very effectively used as imaging agents to amplify tumor microenvironment signals,^173^ but also as nanoparticle vaccines for immunotherapy-based anti-cancer treatments.^174^ The developed PC7A nanoparticles, incorporating an antigen and seven-membered cyclic amine group with a p*K*_a_ of 6.9 (for targeting or early endosomes), were able to induce a strong cytotoxic T lymphocyte response. The system also activates the STING (stimulator of interferon genes) pathway and causes increased and long-term survival in various mouse models of cancer (B16-OVA melanoma, MC18 colon, and a HPV TC1 tumor model). In other non-chemotherapy applications, a collaboration involving Veldhuis, Davis, and Bunnett investigated pH-responsive polymer nanoparticles for the prevention of chronic pain (Figure 10).^175^ By targeting the endosome, the researchers were able to inhibit certain endosome signaling processes (with neurokinin 1 receptor antagonist, aprepitant) and treat chronic pain without using opioids. Systemic administration of the nanoparticle in preclinical mouse models of nociceptive, inflammatory, and neuropathic pain showed increased anti-nociceptive ability, inhibition of spinal neuron excitation, and reduced endosomal signaling compared to the non-nanoparticle formulation. The study highlights an interesting strategy to improve the therapeutic efficacy of antagonists/agonists of GPCRs involved in endosome signaling through use of pH-responsive and endosome-targeting materials.

Polymersomes are biomimetic vesicles, comprised of amphiphilic polymer chains, and offer the ability to encapsulate molecules into nanoscale compartments for drug delivery and imaging.^176−179^ pH-responsive polymersomes were synthesized by Simón-Gracia *et al*. for the delivery of paclitaxel and fluorescent imaging agents to intraperitoneal tumors. The polymersomes were also decorated with the active targeting ligand iRGD or RPARPAR and showed good toxicity against neuropilin-1 (NRP-1)-expressing cells. *In vivo* distribution and efficacy were assessed with mice bearing intraperitoneal tumors from gastric and colon origin, and the paclitaxel-loaded POEGMA-PDPA polymersomes showed significantly reduced tumor growth compared to Abraxane.^180,181^ Salmaso, Vicent, and Alexander developed polymersomes for the delivery of siRNA and nucleic acids to cells *in vitro*. In these examples, the pH response was incorporated through use of an imidazole-containing monomer which mimics histidine with a p*K*_a_ of around 6, which was designed to allow endosome escape and higher intracellular availability of the payload.^182,183^ More recently, Leroux and colleagues demonstrated that pH-responsive polymersomes could also be effective for the treatment of odor-related syndromes.^184^

While the discussed pH-responsive particles are very promising, inorganic and metallic nanoparticles with incorporated pH-responsiveness can incorporate imaging properties or catalytic activity directly into the particle structure.^185−189^ Grafting of polymer chains to (or from) the surface of silica nanoparticles can impart pH-responsive behavior, while silica offers opportunities to include hydrophobic molecules and imaging agents.^190−193^ Zhao *et al*. investigated a theranostic based on hollow silica nanoparticles, which included manganese arsenite complexes and an arsenic trioxide prodrug, thus allowing monitoring of drug release through T_1_ MRI.^193^ By using a pH-dependent complexation strategy, the free manganese ion concentration can be increased in acidic environments, leading to enhanced contrast in the tumor microenvironment. The T_1_ relaxivity value *r*_1_ reached 12.2 mM^–1^ s^–1^ after 8 h at pH 5.4, while it only reached 4.8 mM^–1^ s^–1^ at neutral pH. *In vivo* therapy with this system showed delayed tumor growth in a nude mice human hepatocellular carcinoma tumor model.

When moving to macroscale materials incorporating pH-dependent activity or release, researchers can target a number of different applications more effectively than with nanomaterials. For example, when designing implantable biosensors, conductive and/or fluorescent polymer coatings are an important tool—incorporating pH response can improve functionality.^194,195^ Additionally, certain diseases such as glioblastoma multiforme and other solid tumors require invasive surgeries, leaving large resection cavities. In this case, macroscale materials incorporating endogenous pH response offer an attractive strategy. The Gu lab employed a post-surgical sprayable hydrogel system which incorporated pH-responsive calcium carbonate nanoparticles to modulate tumor-associated macrophage (TAM) behavior *in vivo*.^196^ Encapsulated anti-CD47 antibody is released over time and promotes activation of TAMs to M1-type phenotype, allowing phagocytosis of cancer cells while also increasing antitumor T cell responses. This post-surgical immunotherapy spray inhibited local recurrence and also systemic development of further tumors in a mouse model of incomplete tumor resection and distant tumor.

## Hypoxia

**Figure 11:**
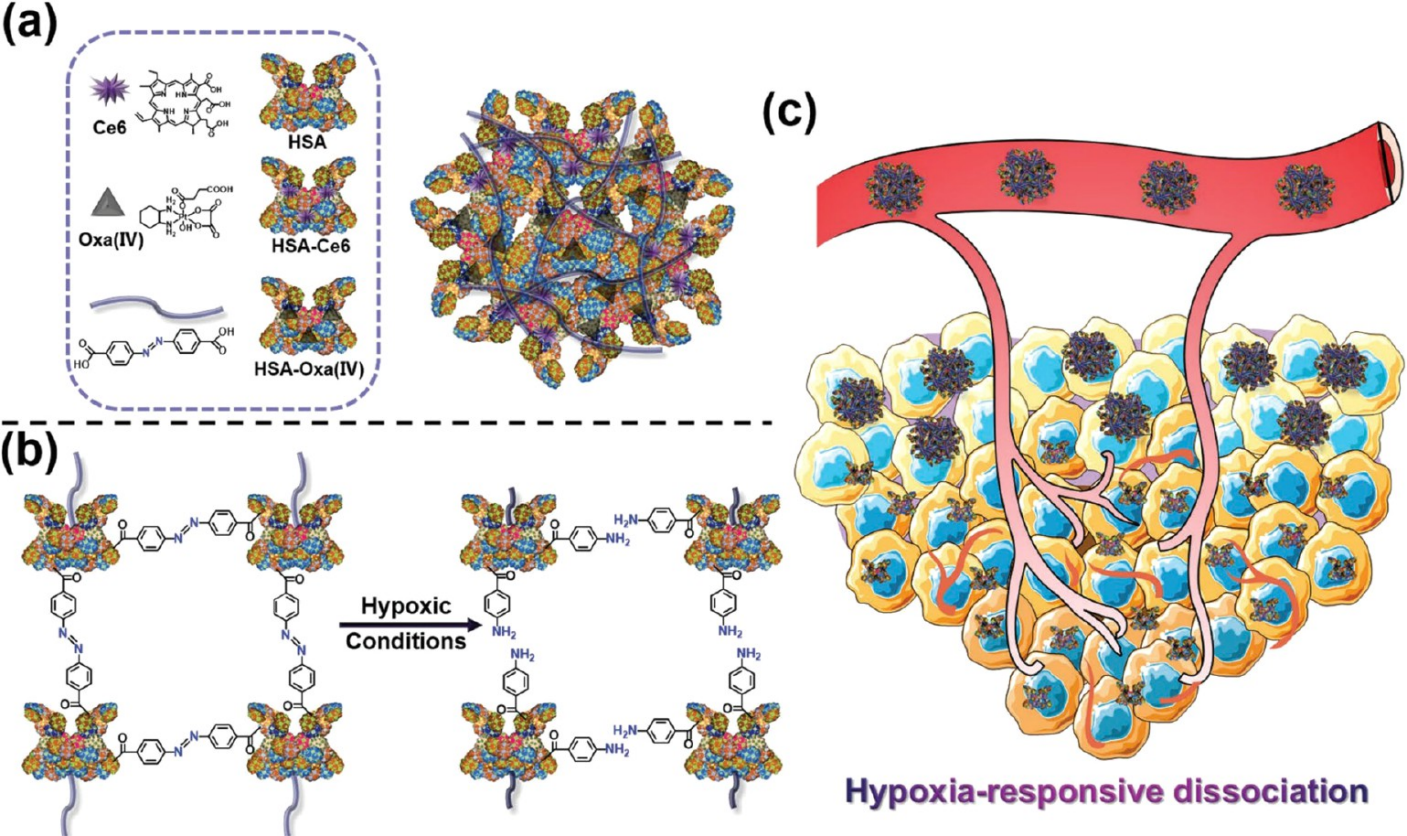
(a) Schematic showing the generation of hypoxia-responsive human serum albumin (HSA)-based nanosystems with photosensitizer chlorin e6, oxaliplatin prodrug, and hypoxia-responsive azobenzene cross-linking groups. (b) Illustration of the hypoxia response mechanism. (c) Tumor activation schematic. Reproduced with permission from ref (202). Copyright 2019 Wiley VCH.

Hypoxia is a condition of inadequate oxygen supply to tissue. It can affect local regions or the whole body and is often associated with vascular diseases, cancer, and ischemia, among other diseases.^197^ In cancer, hypoxia can raise a number of problematic issues for therapy because of its role in tumor progression, restriction of normal tissue function, and potential hypoxia-mediated drug resistance. Quinones are common subunits in various small-molecule active agents and undergo reduction in certain cellular processes, causing semiquinone radical formation and increased production of ROS.^197^ The quinone-based drug mitomycin was identified in the 1960s to incur hypoxia-mediated cytotoxicity, and the moiety can also be included in materials synthesis strategies.^198,199^ Recently, azobenzene has also been used extensively as a unit able to respond to hypoxic conditions for polymersome delivery of gemcitabine to hypoxic pancreatic cancer cells,^200^ for siRNA delivery,^201^ and as an albumin-based nanosystem for tumor theranostics.^202^ Yang *et al*. prepared a responsive particle system by cross-linking the photosensitizer chlorin e6-conjugated human serum albumin and oxaliplatin-conjugated human serum albumin with an azobenzene linker (Figure 11).^202^ This hypoxia-responsive protein hybrid system formed particles of 100–150 nm which were dissociated in hypoxic conditions, leading to deep tumor penetration and increased fluorescence of chlorin e6 for increased imaging sensitivity in the tumor microenvironment. Tumor growth of a 4T1 mouse model was reduced to around 3 mm^3^ (one-third the untreated values) after treatment with the responsive albumin nanoparticles. Fraser and colleagues used a dual-emissive, iodide-substituted difluoroboron dibenzoylmethane group conjugated to poly(lactic acid) (BF2dbm(I)PLA) as a nanoparticle-based sensor for ratiometric tumor hypoxia imaging.^203^ The boron nanomaterials, fabricated using a nanoprecipitation protocol, had excellent fluorescence and phosphorence emissions (λ_F_ = 450 nm, λ_RTP_ = 528 nm) and had sensitivity in the range of hypoxia in biological contexts. The nanoparticles were assessed in a mouse breast cancer 4T1 mammary carcinoma model and were able to produce precise tumor oxygenation maps which could be used with standard tumor optical imaging techniques such as hemoglobin saturation imaging to give vascular information. Researchers have also used the enzyme catalase^204^ and the oxygen-level-sensitive 2-nitroimidazole functional group^205−207^ for a variety of hypoxia-related applications.

## Redox (Glutathione/ROS)

Reduction and oxidation potentials in biological environments offer the opportunity to incorporate a number of redox-sensitive chemistries into imaging and drug delivery systems. Intracellular redox potentials, due to differing levels of glutathione, are exploited to achieve intracellular drug release or triggered imaging while maintaining extracellular stability.^208,209^ Additionally, different pathologies can have drastically different ROS production rates, such as hydrogen peroxide and hydroxide radicals, which can provide a useful trigger for microenvironment-responsive scaffold materials.^210^ These diseases include inflammatory disease, diabetes, cancer, and atherosclerosis.^24^

Cytosol-specific delivery is important for many biologics, including proteins, peptides, and nucleic acids, in immunotherapy and gene therapy, in order to achieve the desired efficacy. Disulfides are covalent bonds able to be reduced to two thiol groups in the presence of a reducing thiol, like glutathione (GSH) or dithiothreitol (DTT). The intracellular concentration of GSH has been reported to be 0.5–10 mM, versus approximately 2 μM in the extracellular space, making it an optimal target for endogenous stimuli responsiveness.^209^ Disulfide-containing dye molecules were employed to quantify cytosolic delivery of biotherapeutics,^211^ and also for the imaging of tumor environments with improved signal-to-noise ratios.^212^ Disulfide moieties are able to be included in many types of polymer nanoparticles.^213−215^ For example, Zhong *et al*. synthesized polymer vesicles from poly(ethylene glycol)-*block*-poly(trimethylene carbonate-*co*-dithiolane trimethylene carbonate)-*block*-poly(ethylenimine).^216^ Granzyme-B, as a protein therapeutic derived from NK cells, was encapsulated in these vesicles, and the surface was functionalized with hyaluronic acid for targeting of overexpressed CD44 in multiple myeloma. *In vitro* the nanoparticles gave a corresponding IC_50_ of 8.1 nM in LP1 human multiple myeloma cells, while *in vivo* treatment of an orthotopic LP1 multiple myeloma model extended survival from 21 to 36 days, simultaneously showing less body weight loss compared to controls. MicroCT imaging documented reduced osteolysis and a decrease in proliferation of abnormal bone marrow cells. Glutathione-responsive cisplatin prodrug nanoparticles were prepared by Ling *et al*. for the treatment of cisplatin-resistant cancers.^217^ In an A2780cis tumor-bearing mouse model of resistant ovarian cancer, the particles reduced tumor growth significantly, which the authors ascribe to the combined effect of intracellular prodrug activation and also the associated minimization of active glutathione, which can reduce the pharmacological activity of cisplatin. Interestingly, there are also numerous examples of disulfide units being incorporated into a range of biological materials, from peptides^218,219^ and lipid materials^220^ to the natural polymers alginate and hydroxyethyl starch.^221,222^

In comparison to reduction-sensitive constructs, oxidation-sensitive materials have applications mostly in inflammation and target oxidative stress, although both involve manipulation of redox homeostasis. In proteins, the amino acid methionine imparts oxidation sensitivity to macromolecular structures and leads to moieties of higher polarity and water solubility. Many synthetic materials mimic this oxidative behavior, such as polysulfides,^223^ polyselenides, oxalates,^189,224^ and thioketals.^225^ Main-chain polysulfides have been extensively described by Tirelli in oxidation-responsive nanomaterials,^226−228^ while slightly more recently, side-chain sulfide polymers have gained increasing attention due to the ability to be polymerized from a variety of different controlled reversible deactivation methods, such as reversible addition–fragmentation chain transfer (RAFT) and atom transfer radical polymerization (ATRP).^229−231^ In the field of theranostics, Zhou *et al*. described an activatable inflammation MRI probe based on nanoparticles formed from the main-chain polysulfide, poly(propylene sulfide).^232^ The approximately 100 nm particles with iron oxide in the polymer vesicle membrane and also gadolinium were used to stratify radiotherapy response early in treatment to better control the therapeutic strategy. Radiotherapy can have variable results among individuals and induces radioresistance and immune response. By employing an oxidation-sensitive MRI probe, the researchers could monitor and stratify tumors based on higher MRI signal from the increasing inflammation. T_1_ relaxation time changes at 24–48 h post radiotherapy treatment correlated with observed immune responses and also tumor growth inhibition following treatment (Pearson’s coefficients *R* = 9.831 and *R*= 9308, respectively). Boronate materials are also employed for endogenous oxidation responsivity, which Satchi-Fainaro and Shabat have shown for combined drug delivery and NIR/optical imaging *in vivo*.^233,234^ The prodrug platform used a QCy7 dye as a central moiety, which was inactive when the hydrogen peroxide-sensitive boronate ester was intact, but under oxidative stress the boronate ester cleaves, activating the dye fluorescence and simultaneously releasing the drug molecule *via* a self-imolative mechanism. Two minutes post-injection in mice bearing U87-MG tumors, the tumor region exhibited a strong fluorescent signal, which continued to emit for 6 h.

Li, Ge, and colleagues have developed a range of amphiphilic block copolymers based on the self-immolative monomer 2-((((4-(4,4,5,5-tetramethyl-1,3,2-dioxaborolan-2-yl)benzyl)oxy)carbonyl)oxy)ethyl methacrylate (BEMA). PBEMA polymers are particularly noteworthy because of their H_2_O_2_-dependent release of quinone methide, which in turn depletes intracellular glutathione levels, suppressing the antioxidative capability of cancer cells.^235−237^ This group of researchers, in collaboration with Kataoka, has also developed a thioketal linker group, which is cleaved by hydroxy radicals (generated by a cascade reaction) and parent drugs thus specifically activated at the site of tumors.^238,239^ These functional groups are elegant examples of interesting chemistries able to enhance therapies by including endogenous oxidation sensitivity, and they should see further use in nanomedicines in the future.

Finally, metal coordination compounds can have interesting redox-responsive behavior, depending on the oxidation state of the metal center. For example, the Wilson group has shown that organic arsenicals can be combined with well-defined polymers of controlled molecular weights for oxidation-responsive nanoparticles and hydrogels.^240−242^ Cobalt can be used as a metal cross-linking agent with redox responsivity.^243^

## Glucose

**Figure 12:**
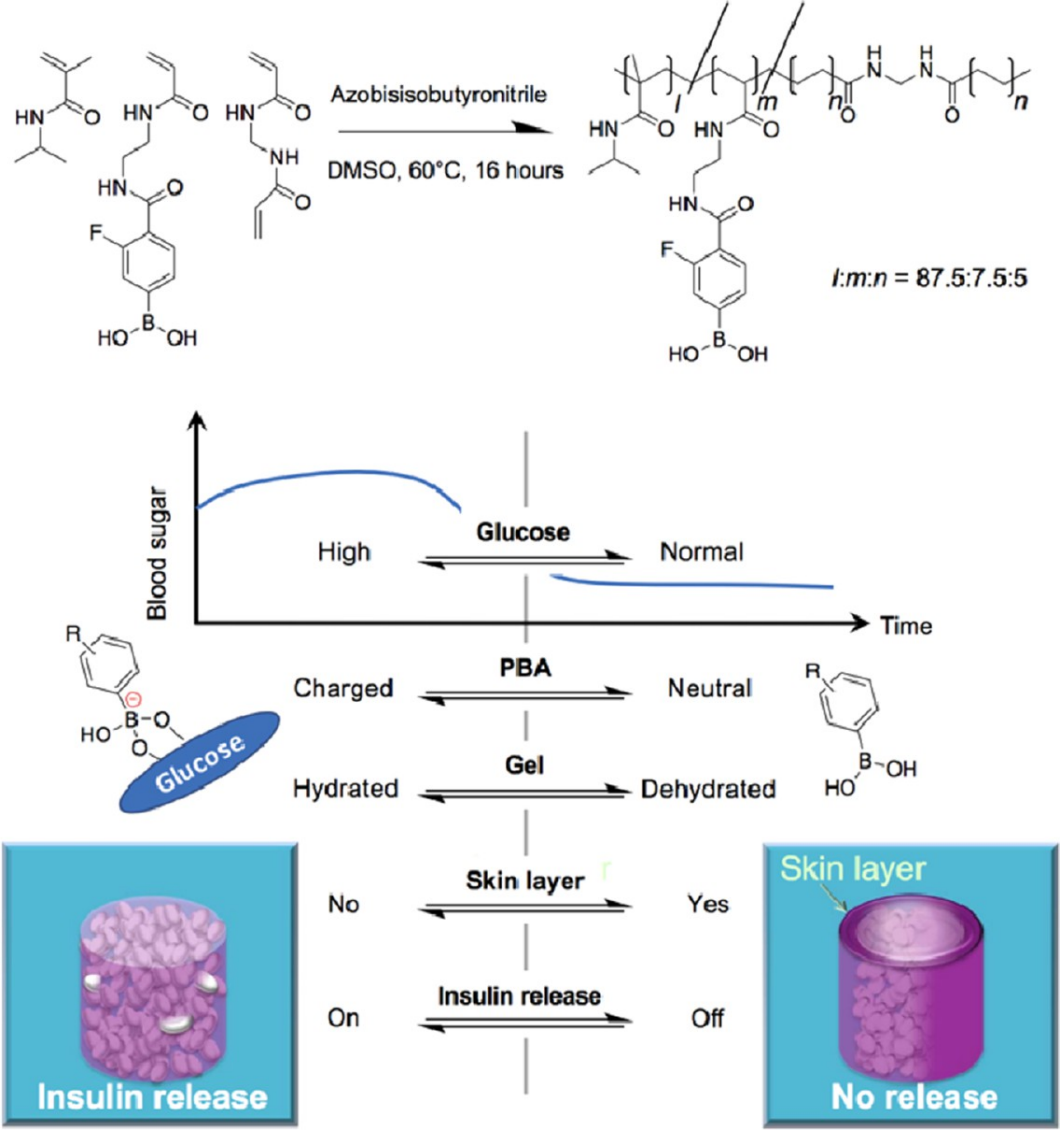
Boronate gel-based insulin delivery system. Schematic showing the chemical structure of the boronate gel-based insulin delivery system and optimal glucose-responsive insulin delivery under physiological conditions (threshold concentration of glucose at normoglycemic (100 mg/dL), above which the gel delivers insulin). Reproduced with permission from ref (253). Copyright 2017 The Authors, some rights reserved; distributed under a Creative Commons Attribution-NonCommercial License 4.0 (CC BY-NC).

Materials and nanoparticles that can undergo a functional or morphological change in response to differing glucose concentrations have applications as hypoglycemia-triggered insulin delivery systems for diabetes patients, and also in pathologies where glucose concentrations are increased due to increased metabolism, such as in cancer.^244−248^ There are two ways researchers typically incorporate this functionality into biomaterials, either *via* phenylboronic acid (PBA) groups or with the enzyme glucose oxidase. The research laboratories of Kataoka and Okano pioneered the use of PBA groups in polymeric materials in the early 1990s,^249−252^ and collaborators continue to push forward the research line today, with improvements in specificity and temporal precision of this molecular recognition system.^253^ PBAs are able to form covalent complexes with a variety of diols, which is the basis of the glucose recognition.^254^ This formation of boronate esters with 1,2- and 1,3-diols is an equilibrium between the unbound and uncharged forms, and the bound and charged forms. Thus, the process is pH dependent, and substitutions on the phenyl ring can improve the binding strength to glucose by altering the p*K*_a_ of the boronic acid. Matsumoto *et al*. fabricated a boronate gel-based delivery material from the free radical polymerization of *N*-isopropylmethacrylamide, *N,N*′-methylenebisacrylamide, and the glucose binding monomer 4-(2-acrylamidoethylcarbamoyl)-3-fluorophenylboronic acid in a porous catheter combined device for subcutaneous implantation (Figure 12).^253^ Implantation of artificial pancreas-inspired device in healthy and diabetic mice allowed interstitial fluid glucose sensing and delivery of insulin from a depot with switchable on/off behavior. After implantation in mice with induced type-1 diabetes, blood glucose concentrations were reduced from approximately 500 to 300 mg/dL. In a type-2 diabetes mouse model, the device also performed well, with slight but statistically significant decreases in average blood glucose concentrations, and other parameters such as C-peptide concentrations were also improved.

The versatility of the PBA group means it can be easily incorporated into a variety of polymeric materials, including nanogels,^255^ polymer micelles,^256−258^ vesicles,^259,260^ and polymer ionic complexes.^261^ The PBA moiety has been used in materials for diverse applications, from glucose sensing and insulin delivery to cancer therapies, shape memory materials, and artificial muscles. Kim and colleagues developed a glucose-responsive helical artificial muscle fiber by coating a helical nylon fiber with PBA-containing hydrogel.^262^ The reversible nature of the bioartificial actuator is achieved through the equilibrium between the untwisting of the core fiber and the recovery force of the polymer coating. Young’s modulus of the fiber was 0.73 MPa in PBS, versus 0.56 MPa in 1 M glucose solution, which the authors assign to be the primary factor regulating the actuation, which is itself due to the change in hydrophilicity of the PBA polymer coating.

**Figure 13:**
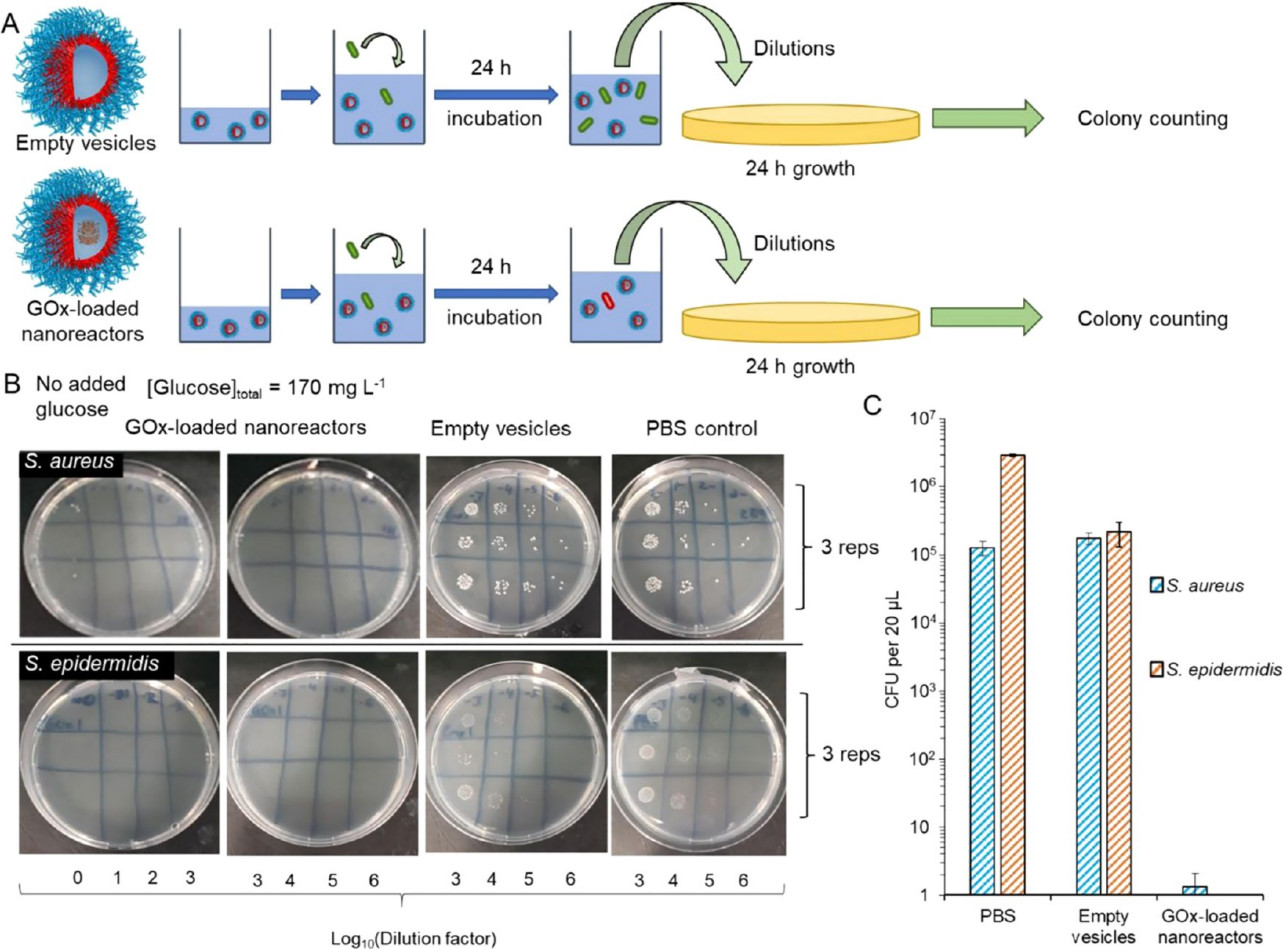
Glucose oxidase-loaded polymer vesicles. (a) Testing protocol for GOx-loaded polymer vesicles and empty vesicles against Gram-positive bacteria. (b) Nutrient agar plates showing associated viability of *S. aureus* and *S. epidermidis* after 24 h incubation. (c) Colony forming units quantification of each bacterial species. Reproduced with permission from ref (263). Copyright 2020 American Chemical Society.

The other alternative component in creating glucose-responsive materials is the enzyme glucose oxidase (GOx), which is used to trigger delivery or morphology changes by sensing components of the GOx catalytic cycle. The GOx enzyme catalyzes the formation of gluconic acid and hydrogen peroxide from glucose, in the presence of oxygen, with a high specificity. A combination of GOx with materials that alter their properties due to changes in products of the GOx cycle (*i.e.*, pH, hydrogen peroxide concentration, or oxygen levels) is used to induce material response. Blackman *et al*. reported antimicrobial and glucose-responsive 220 nm vesicles formed by RAFT polymerization-induced self-assembly (Figure 13).^263^ The formed semi-permeable PEG-*b*-pHPMA nanoreactors could sense glucose through the vesicle membrane, and the entrapped GOx would then produce hydrogen peroxide to give the antimicrobial effect. The switchable activity was confirmed with colony counting, and the nanoparticles could reduce Gram-positive and Gram-negative bacterial growth up to seven-log times. The researchers further optimized the formulation to be non-toxic against human fibroblasts while still maintaining good antimicrobial activity. Similarly, Tirelli and Sommerdijk showed that oxidation-responsive polymer vesicles could encapsulate GOx to achieve glucose-triggered vesicle destruction.^264,265^

GOx-containing systems have been utilized extensively in insulin delivery.^266−268^ Volpatti *et al*. designed and studied acetalated-dextran polymers to control the release of insulin from GOx-containing glucose-responsive nanoparticle formulations.^ref269^ In a streptozotocin-induced type-1 diabetic mouse model, the authors found that subcutaneous injections of 14.4 IU/kg insulin nanoparticle formulation were able to regain glycemic control after being administered in a glucose tolerance test. The nanoparticle treatment group had glycemic control for 16 h from a single dose. The authors concluded that the combination of fast and extended release characteristics was important for self-regulated treatment efficacy. Responsive materials for administration routes such as transdermal are starting to be developed also. For example, the group of Gu has pioneered the use of microneedle devices for insulin delivery.^269,270^ In the field of oncology, glucose-responsive materials have additionally seen use. Cancer starvation therapies (and starvation plus chemotherapeutic combination therapies) have been investigated by researchers as a glucose-responsive cancer treatment strategy.^271−274^ The responsive units of PBA and GOx have also been widely implemented in various clinically approved and pre-clinical electrochemical, fluorometric, and flow-based biosensors for use in point-of-care continuous glucose monitoring devices.^275,276^

## Adenosine Triphosphate

Cell metabolism and appropriate regulation of ATP are fundamental to many cellular processes such as muscle contraction, neurosignaling, and intracellular chemical synthesis. For this reason, ATP is found in high concentrations intracellularly and at much lower concentrations in extracellular environments. Researchers have therefore found it to be an attractive target to trigger intracellular activation of imaging agents and also drug/biological molecule release.^277^ A variety of chemical moieties can be harnessed to build ATP response into nanocarriers and other materials. Competitive binding of ATP to metal complexes and poly(amino acids) has been utilized for theranostic nanoparticles^278^ and transient stomatocyte nanosystems.^279^ Phenylboronic acids can be utilized to bind ATP by forming reversible covalent esters with 1,2- or 1,3-diols which can be found on the ribose ring of ATP (in addition to the glucose-responsive applications of PBAs discussed in the previous section). Materials containing PBAs for drug delivery and imaging include polyionic complex micelles for siRNA delivery,^280^ nanogels for anti-cancer applications,^281^ and conjugated polymer nanoparticles for theranostics.^282^ ATP-sensitive aptamers can be integrated into bioresponsive delivery systems.^283,284^ Zhang *et al*. utilized ATP-sensitive aptamers in a self-assembled polymer nanoparticle formulation comprised of DOX-conjugated poly(ethylene glycol)-poly(aspartic acid) complexed with a hybridized ATP aptamer.^285^ Intracellularly, the ATP will bind to the ATP aptamer and destabilize the nanoparticles. The plasma concentration of DOX *in vivo* for the nanoparticle system was over 7-fold improved (area under the pharmacokinetic plasma curve) compared to free DOX. In an MDA-MB-231 breast cancer xenograft model, the particles showed reduced tumor growth (∼3 × 10^6^ p/s/cm^2^/sr) compared to free DOX (∼5 × 10^6^ p/s/cm^2^/sr), while healthy body weights were maintained over the course of the study.

## Nucleic Acids

The biological macromolecules nucleic acids, including DNA and various RNAs, are possibly the most specific of biological triggers able to be harnessed in synthetic systems.^286−288^ Due to these genetic materials playing important roles in many disease processes and increasingly accessible modern sequencing and synthesis methods, researchers are starting to employ nucleic acid sequences as responsive theranostic triggers.^289,290^ Materials design characteristics, which incorporate dynamic non-covalent responsive behavior due to nucleic acids, can range from nanometer scale to macroscopic hydrogel materials.^291^ For example, Stupp and colleagues synthesized peptide amphiphiles bearing DNA strands that self-assembled into macroscopic hydrogels, with nucleic acid strand-responsive transitions from micelles to long interconnecting fibers.^292^

Nucleic acids are employed in programmable fluorophores to create dyes that interact with certain cells and tissues with a high specificity. Jungmann *et al*. used this to conduct super-resolution microscopy of specific biomolecules of interest *in vitro*.^293^ In diagnostics, RNA- and DNA-coated nanoparticles are reported as genetic RNA detection materials, such as in live *Hydra vulgaris* (a model organism), without affecting the animal’s integrity,^294^ and also for SKBR3 cells *in vitro*.^295^

**Figure 14:**
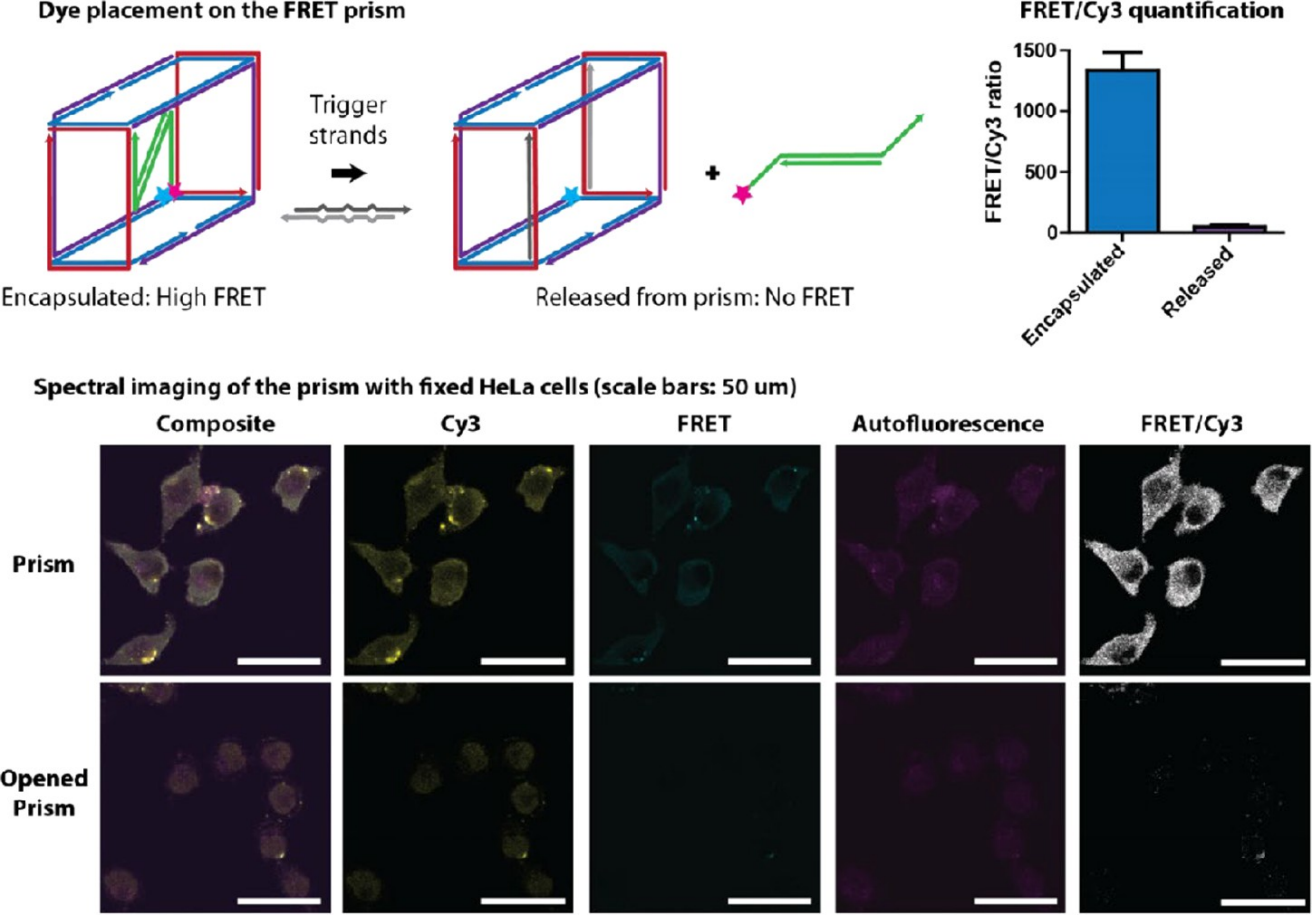
Nucleic acid-based container device. Schematic of the design, assembly, and properties of the nucleic acid origami container device, including FRET quantification of the Cy3 ratio before and after cargo release and images obtained of the nucleic acid system incubated with cells before and after cargo release. Reproduced with permission from ref (297). Copyright 2016 American Chemical Society.

Further to diagnostics, innovative constructs fabricated from nucleic acid strands can assemble into containers and disassemble upon recognition of a particular complementary nucleic acid strand to release their cargo. The Church lab showed this was possible in 2012 with an elegant example of logic-gated molecular transport with tissue cultures.^296^ A DNA origami computer tool was employed to create a barrel-form nanorobot with a size of 35 nm × 35 nm × 45 nm. Antibody fragments or gold nanoparticles could be loaded in the DNA container, with loadings of at least two cargo per container. Fluorescent payloads and flow cytometry were used to determine function. These DNA nanorobots responded to cellular triggers and released their payload to either arrest NKL cell growth or activate signaling pathways of T-cells. Sleiman and colleagues showed that similar DNA cage structures could be used to encapsulate siRNA and achieve conditional release on exposure to oligonucleotide triggers Bcl-2 and Bcl-xL (anti-apoptotic genes), as shown in Figure 14.^297^ Using Cy3 and Cy5 dyes and FRET imaging, the authors showed that siRNA could be released intracellularly, with a 3-fold decrease in FRET signal. Willner and colleagues have also reported a variety of studies on the fabrication and application of microcontainers incorporating nucleic acid strands as responsive motifs.^298,299^ Although nucleic acid-responsive materials as diagnostic tools or therapeutic formulations that perform *in vivo* are complex systems requiring accurate characterization, the opportunity for precise programming of behavior makes them attractive targets for future research.

## Selecting an Appropriate Endogenous Stimulus

Choosing a specific endogenous stimulus for triggering the release of a therapeutic agent or imaging enhancement should consider the type of pathology, the biological site of interest, the chemical complexity of the resulting theranostic system, and the cost of the intervention. Some enzymes are disease-specific, such as the matrix metalloproteinases, extensively used in cancer and cardiovascular diseases; the serum proteases plasmin and thrombin, mostly demonstrated in bone tissue regeneration and thrombotic diseases; and the lipases secreted by bacteria in infected wounds. Other enzymes are more intracellularly localized, such as the cathepsin class of enzymes, and have been exploited to modulate intracellular activities, and some enzymes are more abundant in certain specific organs, like trypsin, efficiently used for intestinal delivery, and esterases, used for activating pro-drugs and nanoparticles in liver. In general, enzyme-based endogenous stimuli are highly specific and can be readily tailored to target a disease or biological site. Moreover, the integration of enzyme-cleavable peptide bonds within the main polymeric structure of the injectable or implantable system can often be achieved straightforwardly. Nonetheless, cleavable peptides are also expensive, even at the laboratory research level.

On the other hand, pH-responsive materials tend to be cheaper, as their response to the biological stimulus is regulated by the presence of simple charged chemical moieties, such as amines or carboxylic acid groups. However, this simplicity is accompanied by a lower specificity. Although local pH variations are indicative of various pathologies (cancer and inflammation), they also occur under physiological conditions in different body districts (stomach and intestine) and intracellular locations (endosomes and other organelles).

Another disease-specific endogenous stimulus is glucose. This has been extensively used in the management of diabetes, as the increased blood content of glucose can be harnessed to control the release of insulin. In this context, PBA groups or glucose oxidase have been employed to modulate drug release or as sensors in diagnostic devices. Glucose content is also altered in malignant tissues, where cancer cells avidly ingest glucose for their proliferation. However, a very limited number of examples are available of glucose-sensitive systems for cancer theranostics.

Redox-sensitive chemistries can be integrated into nanoparticles and polymeric scaffolds to leverage reductive and oxidative environments. Disulfides have been used to respond to the naturally high intracellular concentrations of glutathione to trigger the release of therapeutic and imaging agents. On the other hand, oxidative environments occur at the tissue level during inflammation. Polysulfides, polyselenides, oxalates, and thioketals as well as some metals have been shown to respond to oxidative stresses.

Far less exploited strategies are based on hypoxia and the differential concentration of ATP. The lower content of oxygen is mostly associated with vascular diseases, cancer, and ischemia. Chemical unit such as azobenzene, enzyme catalase, and 2-nitroimidazole functional groups have been integrated in the structure of theranostic agents to trigger the reaction to hypoxic conditions. The actual application of these systems has been limited mostly to cancer. On the other hand, ATP has a high intracellular concentration, and a variety of chemical moieties have been proposed to build ATP-responsive materials that could be activated upon cell uptake. These moieties include PBAs and ATP-sensitive aptamers.

A more recent and scientifically fascinating strategy relies on building nucleic acid sequences that can be included in materials and generate intracellular responses to highly specific genetic information. Indeed, this high biological specificity is balanced by the complexity and cost of the resulting systems.

It is here important to highlight that the response of materials to endogenous stimuli depends not only on the type of stimulus but also on its prevalence. In other words, complex materials design integrating highly specific biochemical sensors could simply fail because the concentration of the endogenous stimulus is insufficient. The strength of an endogenous stimulus varies temporally and spatially, in a complex and often unpredictable manner, as it depends on multiple factors, including the disease type and stage of development, the biological site, and, indeed, the specific patient.

## Barriers to Clinical Translation

The translation of endogenous stimuli-responsive drug formulations and imaging agents to commercial and clinical settings is not simple. Table 2 provides a summary of the clinical trials involving endogenous stimuli-responsive systems. Nanoparticle-based delivery systems are indeed seeing more success reaching the clinic (see recent cases of Vyxeos and Hensify).^300,301^ Including endogenous stimuli response for improved spatiotemporal control can increase complexity and thus also increase potential barriers to translation. The recently approved example of Onpattro is potentially a positive outlier in this scenario, if we consider the ionizable lipids to be endogenously pH-responsive. Historically, various endogenous stimuli-responsive nanodrugs have come close to regulatory approval. For example, from the late 1990s to mid 2000s, cathepsin-responsive pHPMA doxorubicin conjugates PK1 and PK2 reached phase I/II clinical trials, yet did not progress to further clinical use.^303^ As described in the Local pH Variations section above, Arrowhead Pharmaceuticals developed the dynamic polyconjugate system based on a dual pH- and GSH-responsive amphiphilic polymer, poly(butylaminovinyl ether) (pBAVE).^127^ This polymer conjugate, with a number of different therapeutic cargos, completed phase II clinical trials. However, five programs involving the dynamic polyconjugate system were halted in 2016 due to observed high toxicity in primate studies.^304^ Recently, the phospholipase A2 enzyme-responsive candidate LiPlaCis, developed by Oncology Venture, entered phase III clinical trials.^137,305^ The liposomal cisplatin formulation for the treatment of metastatic breast cancer degrades more rapidly in the tumor microenvironment due to overexpression of the target enzyme. Further enzyme-responsive VC peptide linkers for antibody–drug conjugates have already entered the market. Adcetris was approved in 2011 and provides intracellular drug release for the treatment of refractory Hodgkin lymphoma and systemic anaplastic large cell lymphoma.^104^ Thiomabtm, an approved antibody–antibiotic conjugate, uses the same cathepsin cleavable linker for systemic infection therapy.^105^

**Table 2:** Summary of Endogenously Stimuli-Responsive Therapeutics in Currently or Previously in Clinical Trials or Approved for Market

stimulus	target	carrier type	company	phase
**pH**	solid tumors	Nc-6300, polymer micelle, hydrazine-linked epirubicin	NanoCarrier	I
	hepatitis B virus, renal carcinoma, cardiovascular	dynamic polyconjugates, pH-responsive PEG shielding, disulfide siRNA linker	Arrowhead Pharmaceuticals	II (discont. 2017)
	TTR-mediated amyloidosis	Onpattro, siRNA lipid nanoparticle, ionizable lipid DLin-MC3-DMA	Alnylam	approved 2019
	leukemias and carcinomas	BR96-DOX, chimeric monoclonal antibody BR96, linked to doxorubicin through hydrazone linker	Seattle Genetics	II (discont. 2005)
	colorectal cancer	anti-cancer drug Cetuximab, ethylcellulose pH-responsive polymer nanoparticles for oral delivery	Al-Azhar University	I (recruiting 2020)
	head and neck cancer	AP5346, cytotoxic diaminocyclohexane–platinum HPMA polymer conjugate with pH-sensitive linker	PlasmaTech Biopharmaceuticals	II (discont. 2011)

**redox**	acute myeloid leukemia	Gemtuzumab ozogamicin (Mylotarg), monoclonal antibody anti-CD33, disulfide and hydrazone linkers, and calicheamicin drug	Pfizer/Wyeth	approved 2000, withdrawn 2010, re-approved 2017/2018
	lymphoblastic leukemia	Inotuzumab ozogamicin (Besponsa), a monoclonal antibody anti-CD22, disulfide and hydrazone linkers, and a calicheamicin drug	Pfizer/Wyeth	approved 2017
	ovarian cancer	Mirvetuximab soravtansine, humanized anti-FRα mAb M9346A, cleavable disulfide linker, and cytotoxic maytansinoid DM4	ImmunoGen, Inc.	III (recruiting 2020)

**enzymes**				
MMPs	solid tumors	CX-072 enzyme-activatable antibody–drug conjugate	CytomX Therapeutics	II
cathepsin B	solid tumors	PK1, pHPMA-GFLG-Dox conjugate	Pfizer, CRUK	II (discont. 2008)
	solid tumors	PK2, pHPMA-GFLG-Dox conjugate with galactosamine targeting	Pfizer, CRUK	II (discont. 2008)
	solid tumors	PNU166945, pHPMA-GFLG-Paclitaxel congugate	Pharmacia	I (discont. 2002)
	ovarian cancer, lung cancer	Paclitaxel conjugate of poly(l-glutamic acid), lysosomal enzyme-mediated release (CT-2103/Xyotax/Opaxio)	Cell Therapeutics, Inc.	III (discont. 2009)
	lymphoma	Adcetris, Brentuximab vedotin, VC peptide-linked CD30 antibody–drug conjugate	Seattle Genetics	approved 2011/2012
	colorectal, ovarian cancers	CT-2106-degradable conjugate of poly(l-glutamic acid), lysosomal enzyme-mediated camptothecin release	Cell Therapeutics, Inc.	II (discont. 2007)
	*Staphylococcus aureus* infections	DSTA4637S, anti-*S. aureus* antibody linked to antibiotic dmDNA31 through VC peptide linker	Genentech	I (completed 2020)
esterase	breast, ovarian, lung, pancreatic cancers	Genexol-pm, mPEG-PDLLA-formulated Paclitaxel	Samyang Pharmaceuticals	approved 2007 (EMA, South Korea)
	brain metastases	ANG1005 Paclitaxel trevatide	Angiochem	III (recruiting 2020)
phospholipase A2	solid tumor, metastatic breast cancer, prostate cancer, skin cancer	LiPlaCis, cisplatin-containing liposomes, degradable by secreted phospholipase A2	Oncology Venture	III (recruiting 2019)

**hypoxia**	lung cancer	Hypoxia activated prodrug TH-4000 (Tarloxotinib)	Threshold Pharmaceuticals	II (discont. 2017)
	solid tumors	TH302, Evofosfamide, Hypoxia activated prodrug	Threshold Pharmaceuticals/Merck	III (discont. 2015)

**glucose**	diabetes	MK-2640, glucose-responsive insulin saccharide conjugate	Merck	I (discont. 2016)

In general, systems responding to external stimuli have had more successful clinical translation, such as the themoresponsive liposome formulation ThermoDox and the magnetic field-responsive iron oxide nanoparticles NanoTherm. However, in these cases, the stimuli can be externally controlled and monitored. Indeed, one problem that researchers have faced in progressing beyond early clinical trials is the varying expression of these endogenous biological cues in patients, which can lead to sub-optimal efficacy between patients. Therefore, the development of more effective endogenous stimuli-responsive materials should go together with the development of bioanalytical techniques to gather appropriate spatiotemporal information about the prevalence and expression levels of endogenous biomarkers of interest. To help achieve this, further diagnostic tools may be needed to accurately determine distribution and levels of enzymatic biomarkers, pH, and RNA *in vivo* before targeting these stimuli in therapeutic delivery strategies. Inevitably, this would result in more accurate patient stratification and the development of more effective personalized theranostic approaches.^302^

Future efforts should also focus on improving the stability, scalability, reproducibility, and cost of endogenous stimuli-responsive theranostic systems. In this regard, block copolymer micelle nanoparticles are promising candidates due to their scalable synthesis methods.^306,307^ In addition, flow methods for nanoparticle fabrication are also seen as a promising way to surpass some of these problems, with a number of pharmaceutical companies directing significant resources toward flow-based lipid nanoparticle fabrication.^146,308,309^ Of particular concern for endogenously responsive systems is the cost of synthesizing precise peptides, nucleic acid sequences, and other specialty chemicals at the scale needed for commercial application.

## Conclusions and Future Research Directions

Helped by creative advances in synthetic chemistry and fabrication techniques, recent progress in endogenous stimuli-responsive materials has allowed them to be employed in innovative ways, targeting a wide range of pathologies. Despite numerous published successes, the clinical translation of stimuli-responsive nanomedicines and diagnostic tools has been limited to a handful of examples, and for endogenous stimuli this is even less. This highlights the importance of the remaining challenges and helps define exciting future research directions.

In terms of stimuli responsiveness, additional biophysical mechanisms and material design concepts should be explored to realize theranostic systems capable to transition, possibly reversibly, from one morphology to a different morphology rather than undergoing a simple and irreversible assembly/disassembly change. This could lead to an advanced class of bio-inspired theranostic systems. For instance, one could design microscopic particles that change their shape, deform, and cross the hyperpermeable vasculature in an inflamed tissue, activating and responding to the same biological stimuli regulating the diapedesis of circulating leukocytes.

A second particularly exciting research direction, with limited examples so far, is in the design of cascade reactions. By harnessing multiple responsive functionalities, cascade-type transformations could be envisioned for incorporation of multiple and/or synergistic therapeutics in combination therapies and for overcoming sequential biological barriers. One could design hierarchically structured, multi-compartmentalized systems where different endogenous stimuli can be univocally processed to trigger the release of a specific set of molecules and/or imaging agents. For instance, extracellular enzymes could trigger the release of tissue-permeabilizing agents to facilitate the uniform and deep intratissue penetration of a theranostic system. Following cell internalization and endosomal localization, the acidic pH could support the endosomal escape and cytosol accumulation of nucleic acid clusters that would eventually release their therapeutic or imaging cargo based on the genetic phenotype of the targeted cells. Also, utilizing bioresponsive signals that have not yet been employed could enable the targeting of emerging diseases or known diseases with a higher specificity.

A third and fundamental line of research to improve the *in vivo* efficacy of these responsive materials is the accurate identification of the concentration levels of the stimuli. Indeed, their spatiotemporal mapping would help, in a patient- and disease-specific manner, to identify the most relevant stimuli to be harnessed. Use of alternative diagnostic tools will aid this and help to improve outcomes through patient stratification and personalization of treatment regimes. The same stimuli-responsive theranostic systems could be used for generating such a mapping, but they would need to be quantitative and with sufficiently high spatial resolution.

Moreover, expanding the range of material fabrication methods in a promising future area of research. Matching material size, shape, and stiffness to the target organ or tissue can improve patient integration while also giving potential benefits in accumulation. Methods for imparting precise nanomaterial and particle morphologies, such as polymerization-induced self-assembly, two-photon construct fabrication, and 3D printing of materials and biomaterials, including *in vivo* 3D printing of personalized devices,^310,311^ will continue to receive much attention. In summary, inclusion of material physical and chemical changes in response to endogenous biological signals has widened the application of advanced formulations in drug delivery and imaging applications. As researchers continue to find creative applications of these systems, we should see advances in both fundamental understanding of disease pathologies and more effective treatments for a broad range of diseases in the years to come.

## Vocabulary

stimuli-responsive: property or morphology of a material that changes in response to a stimulus such as temperature, light, pH, or the presence of certain (bio)chemical signal.
endogenous stimuli: input stimuli for responsive materials originating from an internal biological source or pathology (compared to an external source, such as radiation)
nanomaterials: natural or synthetic materials with size scales (in at least one dimension) in the range of 1–1000 nm
theranostics: combination of a therapeutic agent with a diagnostic tag or marker in the same formulation, allowing tailored treatment plans or fast evaluation of therapeutic effect
spatiotemporal control: control of active agent delivery with respect to both location and the time frame at which this occurs
